# Landscape characteristics influencing the genetic structure of greater sage-grouse within the stronghold of their range: a holistic modeling approach

**DOI:** 10.1002/ece3.1479

**Published:** 2015-05-01

**Authors:** Jeffrey R Row, Sara J Oyler-McCance, Jennifer A Fike, Michael S O'Donnell, Kevin E Doherty, Cameron L Aldridge, Zachary H Bowen, Bradley C Fedy

**Affiliations:** 1 Environment and Resource Studies, University of WaterlooWaterloo, Ontario, Canada; 2 Fort Collins Science Center, USGSFort Collins, Colorado; 3 US Fish and Wildlife ServiceLakewood, Colorado

**Keywords:** *Centrocercus urophasianus*, functional connectivity, greater sage-grouse, landscape genetics, landscape resistance, mixed models, population genetics, Wyoming

## Abstract

Given the significance of animal dispersal to population dynamics and geographic variability, understanding how dispersal is impacted by landscape patterns has major ecological and conservation importance. Speaking to the importance of dispersal, the use of linear mixed models to compare genetic differentiation with pairwise resistance derived from landscape resistance surfaces has presented new opportunities to disentangle the menagerie of factors behind effective dispersal across a given landscape. Here, we combine these approaches with novel resistance surface parameterization to determine how the distribution of high- and low-quality seasonal habitat and individual landscape components shape patterns of gene flow for the greater sage-grouse (*Centrocercus urophasianus*) across Wyoming. We found that pairwise resistance derived from the distribution of low-quality nesting and winter, but not summer, seasonal habitat had the strongest correlation with genetic differentiation. Although the patterns were not as strong as with habitat distribution, multivariate models with sagebrush cover and landscape ruggedness or forest cover and ruggedness similarly had a much stronger fit with genetic differentiation than an undifferentiated landscape. In most cases, landscape resistance surfaces transformed with 17.33-km-diameter moving windows were preferred, suggesting small-scale differences in habitat were unimportant at this large spatial extent. Despite the emergence of these overall patterns, there were differences in the selection of top models depending on the model selection criteria, suggesting research into the most appropriate criteria for landscape genetics is required. Overall, our results highlight the importance of differences in seasonal habitat preferences to patterns of gene flow and suggest the combination of habitat suitability modeling and linear mixed models with our resistance parameterization is a powerful approach to discerning the effects of landscape on gene flow.

## Introduction

Animal dispersal has important and well-documented effects on population dynamics and persistence (e.g., Vance 1984; Law et al. 2003; Pergl et al. 2011), as well as patterns of diversity and population structure (e.g., Garant et al. 2005; Row et al. 2010). Thus, understanding the factors that influence effective dispersal (i.e., dispersal that results in gene flow) across complex landscapes can reveal the major ecological and evolutionary themes and inform management actions on how to improve or maintain population connectivity. As a result, the last decade has seen the emergence of landscape genetics, which combines landscape modeling with genetic data to better understand how landscape features influence functional connectivity across a given region (Manel et al. 2003; Storfer et al. 2007). Landscape genetics research suggests that genetic models that explicitly consider the influences of natural and anthropogenic features on patterns of population structure can lead to greater ecological insights over those that consider spatial distance alone (Holderegger and Wagner 2008).

The comparison of pairwise genetic differentiation to landscape resistance metrics or cost between any two locations is a common approach to quantifying landscape effects on dispersal (e.g., Row et al. 2010; Munshi-South 2012). If the model fit between genetic and landscape resistance distances is improved compared to a fit with straight-line geographic distance, this suggests a link between the characterized landscape and patterns of effective dispersal (i.e., dispersal that results in gene flow). Although this approach is conceptually straightforward, parameterizing a set of biologically meaningful resistance surfaces and deriving pairwise resistance distances are often challenging in complex landscapes (Rayfield et al. 2009; Spear et al. 2010). This problem is further compounded when considering the added effects of different temporal and spatial scales (Anderson et al. 2010) and accounting for the dependencies inherent in pairwise datasets.

One approach to addressing structural complexity is to synthesize the landscape using multivariate habitat suitability models. We can then use these models to spatially parse the landscape from low (i.e., high suitability) to high (i.e., low suitability) dispersal resistance. Thus, instead of investigating multiple landscape features individually, we transform one biologically meaningful surface and then compare with the genetic data. Generally, resistance values derived from suitability indices have improved the fit over geographic distance, suggesting the distribution and quality of habitat is important in driving gene flow (Laiolo and Tella 2006; Wang et al. 2008; Row et al. 2010). Despite this link, structural connectivity built from habitat suitability indices may not always represent functional connectivity (i.e., gene flow). For example, the habitat used on a daily basis may be different from the habitat a species is willing to travel through when dispersing (Ribe et al. 1998; Spear et al. 2010). Ideally, the comparison of genetic differentiation to resistance derived from habitat suitability indices, as well as individual landscape components, could potentially provide insight into where dispersal and daily-use habitat diverge.

The greater sage-grouse (*Centrocercus urophasianus*; Bonaparte 1827) is distributed across western North America, with a range largely consonant with the distribution of sagebrush (*Artemisia* spp.). With population reductions occurring across the range of sage-grouse (Garton et al. 2011), researchers and land managers are actively studying how to mitigate this decline. Wyoming contains a large proportion of the remaining individuals (>35%; Doherty et al. 2010a) and thus represents a significant component of their current and, likely, future range. A recent large-scale habitat assessment using multiple radio-telemetry datasets was used to derive seasonal (nesting, summer, and winter habitat) resource selection functions (RSFs) for sage-grouse across Wyoming (Fedy et al. 2014). Their derived seasonal habitat suitability maps for sage-grouse highlight the importance of considering a variety of landscape features in evaluating sage-grouse habitat selection. As of yet, there is no assessment of how the seasonal habitat distribution, nor individual landscape components, relates to observed functional connectivity for sage-grouse across this significant portion of their range.

Here, we used linear mixed modeling approaches to compare the importance of individual landscape components and seasonal habitat distribution in driving large-scale patterns of gene flow for sage-grouse across Wyoming. Specifically, we used resistance surfaces transformed to multiple operational scales using differently sized moving windows and parameterized to place an emphasis on variation in low or high resistance to address the following questions: (1) What moving window size and resistance parameterization best characterize functional connectivity for sage-grouse? (2) Is effective dispersal in sage-grouse driven by the distribution of habitat preferences in a particular season? and (3) What is the added value of using habitat suitability indices over individual landscape components in landscape genetics? Lastly, the use of linear mixed models and model selection is a relatively new venture in landscape genetics (Clarke et al. 2002; Pavlacky et al. 2009; Selkoe et al. 2010; Van Strien et al. 2012). Thus, as a final objective we compare and contrast the patterns of four different metrics of model performance and test a method of using standardized regression coefficients (Gelman 2008) to combine resistance surfaces derived from individual landscape components. Overall, our study takes advantage of an extensive dataset to determine the ecological factors driving functional connectivity for sage-grouse within the stronghold of their range and establishes protocols for using mixed models to test dispersal hypotheses across large geographic extents.

## Methods

### Genetic diversity and differentiation

**Figure 1:**
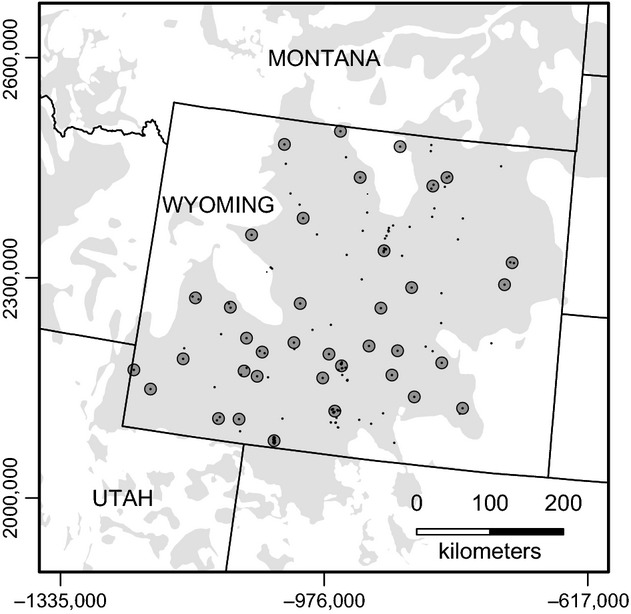
Map of study area delimiting the distribution of genetic samples from the greater sage-grouse (*Centrocercus urophasianus*; Bonaparte 1827) across Wyoming. Black dots represent single or multiple samples, gray transparent circles represent lek buffers (8 km radius) used as grouping in group-based analysis, and light gray polygon is the putative range of sage-grouse across this region. Coordinates for Albers' equal-area projection are displayed.

The Wyoming Game and Fish Department collected feather and blood samples from sage-grouse between the years of 2007 and 2010 across Wyoming and provided these samples for this study. Most of these samples were feathers collected noninvasively and well distributed across lek sites within the state (Fig.1). Details on sample collection and selection, as well as genotyping at 14 microsatellite loci and the identification of unique individuals, are provided elsewhere (See Appendix S1). Our final sample size for the analysis (*n* - 949) represented all unique individuals that amplified at seven or more loci.

The majority of the feathers used in the genetic analysis were collected from leks, and thus, we used these as the basis for defining our groupings (hereafter “lek group”) for pairwise analysis. We do not suggest that our groupings represent biologically unique populations or management units (Waples and Gaggiotti 2006; Palsbøll et al. 2007), but more accurately represent unique sample groups capturing the heterogeneity within Wyoming. We derived our population groupings by first identifying leks with >10 unique individuals and buffering these locations by 8 km, which represents the mean nest to summer range movement distance for sage-grouse (Fedy et al. 2012). Any leks with overlapping buffers were combined, and any individual samples not collected at a lek, but within a buffer, were included with that grouping. This resulted in a total of 612 individuals sampled at 35 lek groups during the breeding season (March–June). This grouping method excluded two regions that had greater than ten clustered samples not collected at leks. In order to include these regions, we added two additional groupings (36 and 37 in Table S2), which contained 43 individuals in total. All of these individuals were sampled in the late summer or fall (August–October), and because average Euclidean movement distance between nesting and late summer is only 8 km (Fedy et al. 2012), we expect these samples to represent breeding populations in the region even though sampling occurred late in the season. Overall, our grouping method resulted in 655 individuals spread among 37 population groupings (Table S1; Fig.1). The dataset was very close to complete with missing data across loci averaging around 2% and the average number of loci for individuals (13.68) being very close to the maximum number of loci (14).

For each lek group, we used the R (R Core Team 2014) package *adegenet* (Jombart 2008) to estimate *H*_exp_, *H*_obs_, and *F*_IS_, and *PopGenKit* (Paquette 2012) to estimate allelic richness with jackknifing (1000 replicates; sample size set to 10). We used the package *mmod* to estimate pairwise differentiation using Nei's *G*_ST_ (Takezaki and Nei 1996) and Jost *D*_est_ (Jost 2008). We also used other differentiation statistics within the *mmod*, but all were highly correlated.

### Resistance surfaces

Although there are many landscape attributes that can potentially influence functional connectivity, we restricted our resistance surfaces to three habitat suitability indices and five major individual landscape components that have the potential to influence connectivity at this large spatial extent. The seasonal habitat surfaces were derived by Fedy et al. (2014), using habitat selection models built with 14 radio-telemetry datasets for sage-grouse. Briefly, the radio-telemetry data were divided by season (NEST, SUMMER, and WINTER) and unique seasonal models were developed across multiple spatial extents and compared using a model selection approach. Here, we were interested in large-scale patterns and thus utilized the top state-wide seasonal models for our analysis (Table1). Details on model development and covariates used can be found in Fedy et al. (2014). The individual landscape component resistance surfaces were derived from percent coverage of forest (FOR), all *Artemisia* sagebrush species combined (SAGE), and agricultural fields (irrigated and nonirrigated; AGRIC), as well as from a terrain ruggedness index (RUGG) and a road decay function from primary and secondary paved roads (ROAD). All of the landscape components were consistent with those used in Fedy et al. (2014) and are known to influence the movement or habitat use of sage-grouse (see more detailed descriptions in Table1). All input layers were originally 30 m^2^ resolution, but due to computational constraints, we resampled them to 300 m^2^ with bilinear interpolation prior to the moving window analysis. Given that seasonal movement distances are typically greater than 10 km, it is unlikely that functional connectivity would be influenced by patterns at resolutions of <300 m^2^. For some input layers, high pixel values represented low resistance to dispersal (e.g., positive predicted effect on gene flow; see Table1) and were reversed by subtracting each value from the maximum value for that surface (Row et al. 2014) and adding 0.1 to avoid zero values (i.e., absolute barriers).

**Table 1:** Resistance surfaces used in sage-grouse (*Centrocercus urophasianus*; Bonaparte 1827) landscape genetic analysis for the state of Wyoming. All resistance surfaces we originally set to 30 m^2^, but resampled to 300 m^2^ resolution before moving window analysis. Any map with a suspected positive influence on gene flow was reversed (max resistance – resistance at each cell) so that all final maps represented resistance with increased values representing higher resistance

Variable	Description of base map	Moving window scales	Predicted effect on gene flow	Source
*Landscape*				
UNDIF	Undifferentiated landscape (i.e., all values set to 1)	NA	NA	
FOR	Percent coverage of forest^1^	1.5 km, 6.44 km, 17.33 km	Negative	Northwest ReGAP
SAGE	Percent coverage of sagebrush (all *Artemisia* species combined)	1.5 km, 6.44 km, 17.33 km	Positive	Homer et al. (2012)
AGRIC	Percent coverage of irrigated and nonirrigated agricultural fields^1^	1.5 km, 6.44 km, 17.33 km	Negative	Fedy et al. (2014)
ROAD	Distance to primary and secondary paved roads. Set up as a decay function (*e*^(*−d*/*α*)^) with *d* as the distance of each raster cell to a road and *α* set to 0.564 km	None	Negative	Fedy et al. (2014)
RUGG	Terrain ruggedness index: range from low values representing flat areas to high values representing steep and uneven terrain	1.5 km, 6.44 km, 17.33 km	Negative	Sappington et al. (2007)
*Habitat Suitability Indices*				
NEST	Nesting habitat suitability derived from resource selection functions^2^	1.5 km, 6.44 km, 17.33 km	Positive	Fedy et al. (2014)
SUMMER	Summer habitat suitability derived from resource selection functions^2^	1.5 km, 6.44 km, 17.33 km	Positive	Fedy et al. (2014)
WINTER	Winter habitat suitability derived from resource selection functions^2^	1.5 km, 6.44 km, 17.33 km	Positive	Fedy et al. (2014)

1 Percent coverage determined from presence in 30 m^2^ cells

2 Used landscape models derived at the state level as described by Fedy et al. (2014)

Each landscape metric, except roads, was transformed using three differently sized moving windows that calculated the average value across the window extent. The radii of the first two moving windows sizes (1.5 km and 6.44 km) represent known regions of influence for habitat selection and movement (Holloran and Anderson 2005; Aldridge and Boyce 2007; Carpenter et al. 2010; Doherty et al. 2010a; Fedy et al. 2012) and are consistent with the derivation of the habitat suitability maps (Fedy et al. 2014). Because dispersal may be driven by processes at larger spatial scales than those that influence habitat selection, a larger moving window size (17.33 km) based on the highest mean interseasonal dispersal distance (nesting to winter range; Fedy et al. 2012) was used for the third moving window.

A species response to resistance in the landscape may not be directly linear. Therefore, for each resistance surface at each spatial scale, we varied the relative strengths of resistance values using two transformations of the original variables. In the equation below, we used an exponential function to effectively homogenize resistances (*R*_*i*_) with low values and place an emphasis on differences in high-resistance habitat,

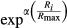
1with *α* being a scale parameter controlling the steepness of the exponential function. Higher values of *α* resulted in greater separation between low and high-resistance values (See Figure S1), and dividing resistance values by their maximum value (*R*_max_) ensured consistency in the transformation between variables with different ranges of resistance values in the original layers (see ranges of untransformed and transformed resistance values in Appendix S2 and S3 as an example). Hereafter, we refer to this transformation as our *high-*resistance transformation.

In the second transformation (hereafter *low-*resistance transformation), we reversed the resistance values (max resistance overall – resistance of cell) and used the resulting values in eq. 1. Subsequently, we reversed these values and added 0.1 to ensure no zero values. This equation results in the converse of the previous transformation and essentially emphasizes variation in the favorable habitats (i.e., low resistance) and homogenizes high-resistance habitats (see Figure S1). We used two different values of *α* (5, 10) for each transformation and thus produced five resistance surfaces for each original surface (untransformed, high 5, high 10, low 5, and low 10; Appendix S2 and Appendix S3). Each transformed surface was summarized across each of the three moving window extents, resulting in 15 resistance surfaces for each landscape variable. Given the low density of class I and II roads across the state, no moving window summaries were conducted for ROAD resulting in only five total resistance surfaces.

We used the derived resistance surfaces to calculate pairwise resistance between groups using CIRCUITSCAPE 3.5.8 (McRae and Shah 2009), which considers the landscape as an electrical network and each cell a resistor with an associated resistance. By running electrical current between nodes (lek groups), the program calculates pairwise electrical resistance (measured in ohms) between locations (McRae 2006; McRae et al. 2008). Current flow and random walkers through electrical networks have a strong relationship (McRae and Beier 2007), and thus, circuit theory has been widely applied to predict patterns of dispersal and gene flow and identify corridors in ecological landscapes (e.g., Schwartz et al. 2009; Row et al. 2010; Moore et al. 2011).

### Model specification

We used a linear mixed model with pairwise genetic distance as the dependent variable and pairwise resistance values as the independent. The model also includes a random effect term that accounts for data points that share a common lek group (coded as 1) and those that do not (coded as 0) (Clarke et al. 2002; Van Strien et al. 2012). Thus, the proportion of total variance associated with the correlation among data points that share a common group is addressed in the model formulation (Clarke et al. 2002). The models were first estimated with the *lmer* package (Bates et al. 2014) in R (R Core Team 2014) using REML, which is required to obtain unbiased estimates of the variance (Clarke et al. 2002; Van Strien et al. 2012). Secondly, the models were estimated using the *MCMCglmm* package (Hadfield 2009) with a similar model formulation for the random effects term *(˜idv(mult.memb(˜pop1 + pop2)*)). We assessed convergence of the MCMC models by comparing results across multiple runs. Estimates from *lmer* and *MCMCglmm* were nearly identical, and thus, we present coefficients from *lmer*, but confidence intervals from *MCMCglmm*. Because we were interested in determining the relative influence of each landscape metric, variables were standardized and centered around their mean (Gelman 2008) for all models.

### Model selection

We selected the top models through a combined assessment of four model selection criteria. We calculated AIC_*c*_ from the *lmer* models and DIC from the *MCMCglmm* models. Although information criteria (including AIC) are generally regarded as not appropriate for models estimated with REML (Verbeke and Molenberghs 2000), this metric has been used for model selection in landscape genetics (Selkoe et al. 2010; Richardson 2012) and demonstrated as potentially informative with REML through simulation (Gurka 2006).

Secondly, we used two marginal *R*^2^ values to estimate the amount of variation explained by the fixed effects. Marginal *R*^2^ values compare a model with only the random effects (e.g., the model term accounting for the pairwise comparisons) to models with random and fixed effects. Unlike traditional *R*^2^, marginal *R*^2^ can decrease with the addition of variables (Van Strien et al. 2012). The first metric 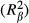 quantifies the difference in explained variation between the models with and without fixed effects using the F-distribution (Edwards and Muller 2008), which we estimated using Kenward–Rogers' approximation as implemented in the *pbkrtest* R package (Halekoh and Højsgaard 2011). The second metric we used was 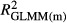, as defined by Nakagawa and Schielzeth (2013), where the fixed effects variance is estimated by calculating the variance of fitted values predicted from a model with only fixed effects. In contrast to 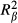, there is no correction for the degrees of freedom. Both marginal *R*^2^ statistics provide a measure of the variance explained by the fixed effects with higher values indicating better model fit.

There is no clear preferred method of assessing model fit within the context of our research, as there are uncertainties associated with all of the criteria discussed above. Thus, we also calculated a mean model rank (i.e., for all criteria combined) by ranking the candidate models separately for each criteria and then determining the mean rank and standard deviation across models. This provided a consensus value (mean) and an estimate of agreement (standard deviation) between the different model selection criteria.

### Ecological determinants of functional connectivity

#### Spatial scale and resistance parameterization

In total, we had 15 resistance surfaces for each landscape variable (three habitat indices and five landscape components; Table1). All of the 15 surfaces varied in moving window sizes and/or parameterization (i.e., exponential transformation), and for each, we fit a univariate model of genetic differentiation to the derived set of pairwise resistances. The most appropriate spatial scale and resistance transformation for each variable were selected by choosing the model with the best (i.e., lowest) mean model rank. The top resistance values for each component and index were used in the model sets described below.

#### Dispersal hypotheses

We derived a set of 22 models that describe resistance to dispersal for sage-grouse across Wyoming (Table2). As per our objectives, we designed the set of models to compare the ability of seasonal habitat suitability indices (HSI models; models 2–4) and individual and combined landscape components (component models; models 5–22) to describe functional connectivity. We compared models and their variables by comparing the individual and combined model selection indices and the standardized regression coefficients and their confidence intervals.

**Table 2:** Multivariate models used to describe functional connectivity for sage-grouse (*Centrocercus urophasianus*; Bonaparte 1827) across the state of Wyoming

Model ID	Model
1	*GEN˜UNDIF*
2	*GEN˜NEST*
3	*GEN˜SUMMER*
4	*GEN˜WINTER*
5	*GEN˜FOR*
6	*GEN˜FOR+RUGG*
7	*GEN˜FOR+ROAD*
8	*GEN˜FOR+ROAD+AGRIC*
9	*GEN˜FOR+AGRIC*
10	*GEN˜SAGE*
11	*GEN˜SAGE+RUGG*
12	*GEN˜SAGE+ROAD*
13	*GEN˜SAGE+ROAD+AGRIC*
14	*GEN˜SAGE+AGRIC*
15	*GEN˜FOR+SAGE*
16	*GEN˜FOR+SAGE+RUGG*
17	*GEN˜FOR+SAGE+ROAD*
18	*GEN˜FOR+SAGE+ROAD+AGRIC*
19	*GEN˜FOR+SAGE+AGRIC*
20	*GEN˜ROAD*
21	*GEN˜ROAD+AGRIC*
22	*GEN˜AGRIC*

#### Combined landscape components

An advantage of using habitat suitability indices in landscape genetic models is that the result is a single resistance surface that researchers can incorporate in other applications such as the identification of dispersal corridors. In contrast, a model selection approach may select a set of best-fit multivariate models with the pairwise resistance values, but not generate a single resistance surface that can be used in other applications. We attempted to address this problem by devising a single landscape resistance surface using the coefficients derived from the top multivariate models. Because all variables were standardized, we determined the relative weights of each variable by dividing the value for each coefficient by the sum all coefficients in the model. Next, we used raster math to multiply these values by their respective resistance surfaces and summed all surfaces (e.g., final raster values - coefficient weight A × raster A + coefficient weight B × raster B) resulting in a single combined resistance surface. We validated this approach by calculating pairwise resistances from the combined surfaces and compared the fit of the resulting pairwise resistances with genetic data. An increased fit of the combined resistance surfaces compared to models with the individual components alone would suggest some value to this approach.

## Results

### Genetic diversity and differentiation

Overall diversity indices were relatively high and consistent between groups with allelic richness ranging from 5.05 to 7.52 and *H*_exp_ ranging from 0.70 to 0.82 (see Table S1). Pairwise genetic differentiation between lek groups ranged from 0.0004 to 0.058 for *G*_ST_ and from 0.004 to 0.66 for *D*_est._ Although there were differences in the magnitude of the values, they were highly correlated (0.98; see Figure S2), and thus, only the results for *G*_ST_ are reported.

### Ecological determinants of functional connectivity

#### Spatial scale and resistance parameterization

Not surprisingly, there were generally strong correlations among resistance surfaces across moving window sizes and among transformations (see Figure S3, Tables S2 and S3). For the habitat indices (NEST, SUMMER, and WINTER) and SAGE, changing the moving window size alone did not have a strong effect on resistance values (see Figure S3). Changing the transformation strength, however, tended to result in greater changes in the level of correlation among differently scaled resistance surfaces, with values as low as 0.6. For the landscape components, which were less continuously distributed in their native form (RUGG, AGRIC, and FOR), scale and strength transformation resulted in similar deviations between resistance surfaces (see Figure S3).

**Figure 2:**
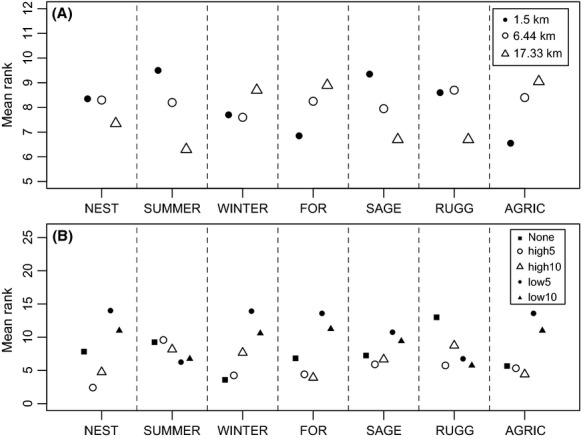
Mean model selection ranks for univariate models describing functional connectivity for sage-grouse (*Centrocercus urophasianus*; Bonaparte 1827) across Wyoming. The scale of moving window (A) and transformation of resistance values (B) for habitat suitability and individual landscape components were varied. Lower ranks are the preferred model.

For NEST, SUMMER, SAGE, and RUGG, resistance values derived from surfaces with a 17.33-km moving window had better ranks overall (Fig.2; Tables S2 and S3), with the top model for all of these landscape metrics being derived from this moving window size (Table3). For FOR and AGRIC, the opposite pattern emerged with the smallest moving window size (1.5 km) having better model ranks (Fig.2; Table3). There was little difference in model ranks for the WINTER surface resistance values, with the top model coming from a 6.44-km moving window (Table3). With the exception of WINTER and RUGG, using the high-resistance transformation led to better model ranks (Fig.2) and none of the top models contained resistance values derived from the low-resistance transformation (Table3). For WINTER and AGRIC, the top model contained no strength transformation, with all other top models transformed to a strength value of High5 or High10 (Table3).

**Table 3:** Top univariate models and associated ranks for habitat and landscape resistance surfaces describing functional connectivity for sage-grouse (*Centrocercus urophasianus*; Bonaparte 1827) across Wyoming

Model	Moving Window	Transformation	Mean rank	SD rank
NEST	17.33 km	High 10	1.75	0.96
SUMMER	17.33 km	High 5	5.5	3.70
WINTER	6.44 km	0	2.75	1.26
FOR	1.5 km	High 10	3.25	3.86
SAGE	17.33 km	High 5	4.5	3.87
RUGG	17.33 km	High 10	4.25	1.5
AGRIC	1.5 km	0	3.25	1.06
ROAD	NA	High 10	1.5	1.00

#### Dispersal hypotheses

Correlation between pairwise resistance values derived from the top habitat resistance maps ranged from 0.69 (SUMMER–WINTER) to 0.84 (NEST–WINTER; Figure S4). Pairwise resistance values from SUMMER had higher correlation with values derived from UNDIF (0.70) than did resistances from the NEST (0.58) and WINTER (0.65) surfaces. In general, the pairwise resistance values were normally distributed (see Figure S4). Correlation among resistances from the top landscape components ranged from 0.48 to 0.83 and between 0.57 (AGRIC) and 0.94 (ROAD) for their correlation with UNDIF values (see Figure S5).

**Figure 3:**
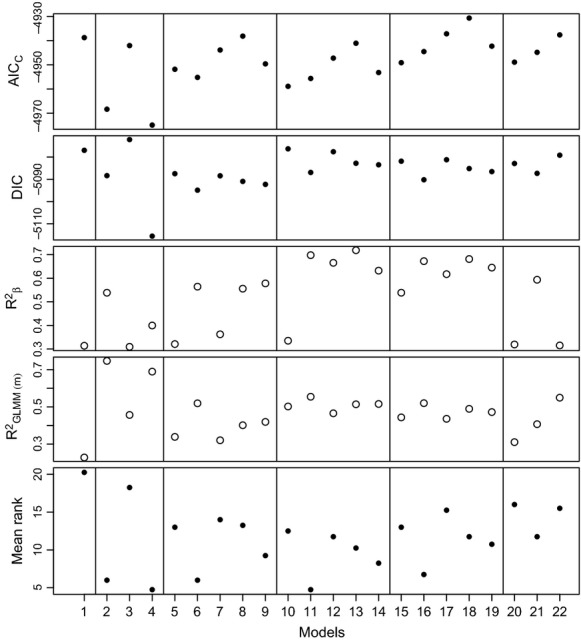
Individual and combined model selection criteria for multivariate models (Table2) relating pairwise genetic differentiation to pairwise resistance derived from different landscape metrics for sage-grouse (*Centrocercus urophasianus*; Bonaparte 1827) across Wyoming. Closed circles represent criteria where lower values should be preferred (better model), while open circles represent the opposite.

Model 1 (UNDIF) was the poorest ranking model. This model also had a low standard deviation in model rank, suggesting it performed poorly for all model selection criteria (Fig.3; Table4). The best consensus ranked HSI models were model 2 (NEST) and model 4 (WINTER), which performed much better than model 3 (SUMMER). This trend was generally consistent across all model selection criteria, with the exception of *R*^2^*β*, which preferred more complex models (Fig.3). The best landscape component model was model 11 (SAGE and RUGG), which had a similar ranking to the best HSI models (Fig.3; Table4). Component models 6, 11, and 16 all had similar ranks, suggesting that FOR, RUGG, and SAGE were all contributing to effective dispersal, but that FOR and RUGG and SAGE and RUGG could adequately capture this trend, and the inclusion of all three (FOR, SAGE, and RUGG) did not improve model fit. The anthropogenic resistance surfaces (ROAD and AGRIC) did not appear to have a strong influence on model rankings, although AGRIC was included in one of the top six models (Table4). Beyond the agreement for the superior fit of NEST and WINTER, there were differences between the model selection indices leading to low correlations between the different criteria (Figure S6).

**Table 4:** Highest and lowest model rankings for multivariate models used to describe functional connectivity for sage-grouse (*Centrocercus urophasianus*; Bonaparte 1827) across the state of Wyoming

Model	Rank	AIC_*c*_	Δ AIC_*c*_	DIC	Δ DIC	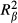	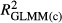	Mean rank	SD rank
*GEN˜WINTER*	1	−4974.89	0	−5115.56	0	0.40	0.69	4.75	6.85
*GEN˜SAGE+RUGG*	2	−4955.65	19.24	−5086.91	28.65	0.70	0.55	4.75	3.59
*GEN˜NEST*	3	−4968.34	6.55	−5088.35	27.21	0.54	0.75	6.00	5.94
*GEN˜FOR+RUGG*	4	−4955.20	19.69	−5094.89	20.67	0.56	0.52	6.00	3.74
*GEN˜FOR+SAGE+RUGG*	5	−4944.54	30.35	−5090.20	25.36	0.67	0.52	6.75	4.19
*GEN˜SAGE+AGRIC*	6	−4953.19	21.7	−5083.47	32.09	0.63	0.52	8.25	3.20
*GEN˜SUMMER*	21	−4942.06	32.83	−5072.12	43.44	0.31	0.46	18.25	4.50
*GEN˜UNIDF*	22	−4938.77	36.12	−5076.96	38.6	0.31	0.23	20.25	1.71

**Figure 4:**
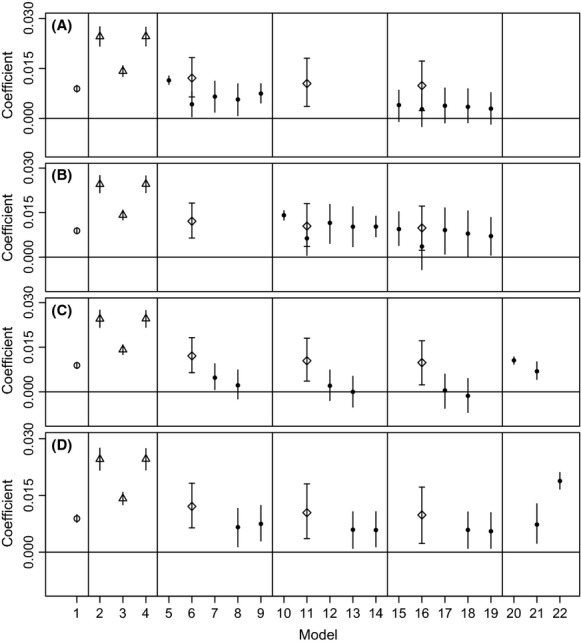
Standardized coefficients and their confidence intervals from sage-grouse (*Centrocercus urophasianus*; Bonaparte 1827) dispersal hypothesis models (Table2) with (A) FOR, (B) SAGE, (C) ROAD, and (D) AGRIC coefficients shown as filled circles. Habitat index coefficients are also shown as triangles, UNDIF is an open circle, and RUGG is shown as a diamond with capped error bars.

Examining relative model coefficients, values for WINTER and NEST were similarly higher than all other coefficients (Fig.4). FOR and SAGE were always significantly positive, except when combined together in the same model, and FOR was often reduced and nonsignificant (Fig.4). RUGG had a higher coefficient than both SAGE and FOR. However, given the overlapping confidence intervals and high correlations between SAGE, FOR, and RUGG, it would be difficult to determine the relative importance of these three surfaces. The coefficient for ROAD was generally nonsignificant when combined with other variables, but AGRIC was significantly positive in all models.

#### Combined landscape resistance

**Figure 5:**
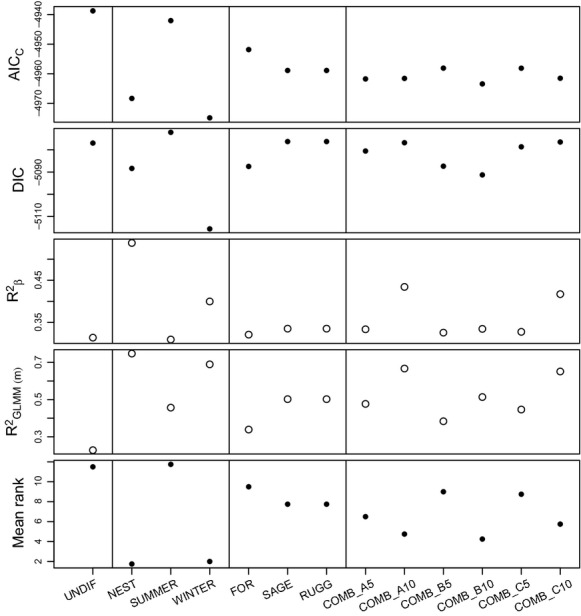
Modeling resistance of combined landscape component resistance surfaces describing functional connectivity for sage-grouse (*Centrocercus urophasianus*; Bonaparte 1827) across Wyoming. Combine A (SAGE and RUGG), combine B (FOR and RUGG), and combine C (FOR, SAGE, and RUGG) with combined landscape components transformed using the high5 and high10 resistance transformation. Closed circles represent criteria where lower values should be preferred, while open circles represent the opposite.

Eliminating the anthropogenic models, which had a much lower fit, one of the top three landscape component models was within each of the remaining model groupings, with a large gap in rank for the next highest ranking model in the group (Fig.3). Thus, these models were fairly representative samples and we determined the relative model coefficient weights for the variables in each of these models (SAGE and RUGG; FOR and RUGG; FOR, SAGE, and RUGG). Because of the differences in parameterization, we could not combine surfaces with different transformations and thus derived combined maps with the High5 and High10 transformed resistance surfaces separately. Overall, the High10 combined surfaces all provided superior fit over the models with individual landscapes, but not the multivariate models or those containing the top HSI surfaces (Fig.5).

## Discussion

Through model selection and parameter estimation, we were able to discern the major ecological factors driving functional connectivity for a mobile terrestrial vertebrate. Overall, the broad-scale (i.e., large operational scale) distribution of high-resistance (i.e., low quality) nesting and winter seasonal habitat appeared most important in driving patterns of effective dispersal for sage-grouse across this spatial extent. Comparing the results of models containing habitat suitability indices and individual landscape components suggested some convergence between dispersal and daily-use habitat, and a benefit (i.e., improved model fit with genetic data) to using habitat suitability modeling in landscape genetics.

### Ecological determinants of functional connectivity

#### Spatial scale and resistance parameterization

The spatial scale with which organisms respond to landscape structure is largely determined through their inherent dispersal ability and sensitivity to changes in a given landscape feature (D'Eon et al. 2002; Anderson et al. 2010). Indeed, studies that have considered varying spatial scales in landscape genetics have demonstrated that the importance of a landscape variable to patterns of genetic structure is dependent on the spatial scale with which it is quantified (Baguette and Van Dyck 2007; Murphy et al. 2010; Wasserman et al. 2010). Although we found that most resistance distances were correlated across different spatial transformations, large-scale patterns appeared most important with resistances transformed using large moving windows (6.44 km or 17.33 km in radius) providing the best fit with genetic data. This is perhaps not surprising as sage-grouse habitat selection is influenced by habitats at large landscape scales (Doherty et al. 2010a; Knick and Connelly 2011; Aldridge et al. 2012; Fedy et al. 2014) and interseasonal movements can exceed 90 km and average as much as 20 km for some populations (Fedy et al. 2012). Thus, small-scale differences in habitat structure are unlikely to have major impacts on the dispersal behavior of sage-grouse across this large spatial extent.

Although we altered the operational scale using moving windows, the spatial extent of our study area remained fixed because we were interested in establishing patterns of connectivity across the entire state. However, for other species the landscape features influencing patterns of gene flow vary depending on their availability (Moore et al. 2011; Short Bull et al. 2011). Indeed, this was the case for habitat selection as Fedy et al. (2014) found the importance of individual landscape characteristics varied among regions within Wyoming, possibly driven by differences in habitat availability. Thus, changing the extent examined here and comparing patterns observed in different regions of the state could provide further insight into the main factors influencing patterns of differentiation for sage-grouse.

In addition to the importance of spatial scale, choosing appropriate and biologically meaningful values for resistance surfaces can be challenging and have an impact on results (Spear et al. 2010). Resistance surfaces should reflect spatial variation in the distribution of landscape features that will impede or facilitate gene flow and hence influence functional connectivity. However, a species response to landscape features is not necessarily summative. We used two different equations to assign resistance values to ecological variables and found that when higher overall values were placed on high-resistance habitats, it generally led to a better fit with genetic data (AGRIC, WINTER, and FOR were exceptions). This pattern is consistent with other studies that have grouped habitat suitability indexes and found that placing exponentially higher resistance values to extremely low-quality habitat led to resistance distances that had a greater correlation with genetic data (Wang et al. 2008; Row et al. 2010). In fact, Row et al. (2010) found when resistance values calculated for very unsuitable habitats were defined as absolute barriers, these surfaces provided the best fit with genetic data. These results appear to suggest that for dispersing individuals, the quality of usable habitat is less important than the distribution of very unsuitable habitat, but more studies are required to determine whether this pattern is consistent across species and regions.

#### Dispersal hypotheses

Landscape genetic studies are increasingly utilizing habitat suitability indexes to derive resistance surfaces. In the majority of these studies, least-cost paths or resistance distances derived from habitat suitability offer a better fit with genetic data than Euclidean distance (Wang et al. 2008; Row et al. 2010; Shanahan et al. 2010; Shafer et al. 2012; Razgour et al. 2014), which highlights the importance of habitat suitability driving dispersal. Here, we took the additional step of utilizing seasonal habitat models, which offered unique insights into sage-grouse dispersal biology. We found that habitat preferences for particular seasons were more important in driving patterns of genetic differentiation. Adult sage-grouse typically display high fidelity to their seasonal distributions and breeding sites (Berry and Eng 1985; Schroeder and Robb 2003; Fischer et al. 2013). Thus, it follows that overall dispersal patterns in sage-grouse should be driven by yearling sage-grouse searching for suitable nesting habitat upon leaving their initial wintering sites, perhaps leading to our finding that the distribution of nesting and winter habitat was more important than summer habitat for functional connectivity. Regardless of the mechanism, these seasonal differences and the fact that dispersal rates can vary depending on life stage (Dobson 1982), season (Southern 1962; Long et al. 2008), or the current demographics of a population (Poole 1997) highlight the importance of considering dispersal biology when devising resistance surfaces for landscape genetics.

In addition to the distribution of seasonal habitat, some individual landscape components appeared more important for functional connectivity. Positive associations with sagebrush and negative associations with forest and terrain ruggedness are common to habitat selection studies on sage-grouse (Carpenter et al. 2010; Doherty et al. 2010a; Aldridge et al. 2012; Fedy et al. 2014). We found these features were not only common to one (terrain ruggedness included in winter, but not nesting) or both (forest and sage included in both HSIs) of the top HSI models, but were also present in the top component models demonstrating their overall importance to functional connectivity. Despite the apparent importance of these variables, the inclusion of SAGE and FOR in the same model did not improve model fit. This is likely because they are negatively correlated on the landscape at large spatial scales and the impedance of gene flow from forests and the facilitation of gene flow from sagebrush likely have similar effects on overall genetic structure.

A direct agreement between the HSI models and landscape component models was not always the case. Both major roads (Aldridge and Boyce 2007; Atamian et al. 2010; Carpenter et al. 2010; Doherty et al. 2010a; Dzialak et al. 2013) and agricultural fields (Aldridge and Boyce 2007; Walker et al. 2007; Fedy et al. 2014) have proven to be negatively associated with habitat selection, and both landscape components were included in the winter and nesting HSIs. Neither component, however, was present in the top component models, suggesting they were not having strong overall effects at this spatial extent. This was particularly true for roads, which were not included in any of the top models and had coefficients that overlapped with zero for most of multivariate models. It should be noted, however, that resistance values from roads were highly correlated with an equal landscape (0.94). This is likely because there are relatively few class I and class II roads (i.e., paved) across Wyoming leading to minimal variation in the number of roads that are crossed between pairwise locations. In contrast, the resistance values from AGRIC had one of the lowest correlations with an equal landscape (0.57) and the coefficients for AGRIC were significantly greater than zero for all multivariate models. In addition, AGRIC with SAGE performed relatively well when compared with other component models suggesting a potential negative effect of agricultural fields on sage-grouse connectivity. Given the negative association between sage-grouse habitat selection and these anthropogenic landscape features and their uneven spatial distribution on the landscape, it may be particularly important to conduct analysis at smaller spatial extents before drawing conclusions on their overall impact on functional connectivity.

Across our study region, there are two mountain ranges (Big Horn and Wind River) that likely limit sage-grouse dispersal. This is evidenced by the fact that all of the resistance values that had the best fit with genetic data were derived from surfaces that placed high-resistance values to these regions. Given the extent of these mountain ranges, these large-scale barriers may have a disproportionate influence and mask the effect of some of the other landscape features (e.g., roads, agricultural fields), which may have more subtle impacts. Future studies conducted at smaller spatial extents could test for regional variation in the patterns observed here and make valuable contributions to our understanding of the influence of varying spatial extents on our conclusions. Further, the importance of individual landscape variables can vary with their availability (Short Bull et al. 2011), and thus, it may be equally important to determine whether different patterns emerge when conducting similar studies in regions with lower amounts of high-quality habitat or greater habitat fragmentation.

### Use of model selection approaches in landscape genetics

Mixed models are an important tool to account for dependence in pairwise datasets in landscape genetics (Pavlacky et al. 2009; Selkoe et al. 2010; Van Strien et al. 2012). As presented here, mixed modeling approaches allow for the comparison of a model set that can represent a suite of biological hypotheses about the functional connectivity of wildlife populations. Despite the potential value of combining mixed models with model selection, the different model selection criteria used here did not always agree on model rank. Van Strien et al. (2012) suggested using 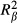 to select the top mixed model, but when comparing with the other selection criteria, it appears this criterion was biased toward more complex models. 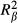 was the only criteria that did not select one of the HSI models as the top model and instead selected a component model with three parameters. In addition, the top three models chosen by 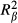 had two or more parameters, and all of the lowest ranking models had a single parameter. This could have important consequences when comparing multivariate and univariate models together. 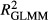 was more in agreement with AIC_c_ and DIC and had higher values for single parameter models (WINTER and SUMMER). Overall, these results indicate that there needs to be more research on the most appropriate model selection criteria for different circumstances. The selection criteria may vary in their accuracy when attempting to explain different processes and sampling variation. In the absence of a clearly preferred criterion, we recommend the assessment of agreement across multiple selection criteria as presented here.

A potential disadvantage of using model selection approaches is that although the approach will establish a model describing functional connectivity, it will not lead to an overall resistance surface if the top model is multivariate. We attempted to circumvent this problem by using standardized regression coefficients that combine individual resistance surfaces into a combined surface. In general, pairwise resistances derived from the combined surfaces provided a better fit than values derived from a single landscape component, suggesting that this may be a promising approach. Despite this improvement, the level of fit with genetic data was not as great as the multivariate models or the HSI models, and thus, more research is required into the best methods for translating multivariate models into individual resistance surfaces. In the meantime, our results combined with other studies linking HSIs and genetic differentiation (Laiolo and Tella 2006; Wang et al. 2008; Row et al. 2010) suggest combining habitat suitability modeling with landscape genetics may be a useful approach when a single resistance surface is desired.

### Conservation implications

Across their range, sage-grouse have experienced population declines and range contractions (Schroeder et al. 2004; Garton et al. 2011). In recognition of this, core areas within Wyoming have been identified and prioritized for sage-grouse conservation (Doherty et al. 2011), yet there is little understanding of how well connected those areas are and whether they will stay connected into the future. Here we provide the first formal assessment of functional connectivity for sage-grouse in Wyoming and establish the importance of seasonal habitat indices and individual landscape components in promoting (sagebrush) and impeding (forest, terrain ruggedness) gene flow. Further, we have established several resistance surfaces that describing functional connectivity for sage-grouse, which can be used and manipulated to identify and protect regions disproportionately important for maintaining connectivity between existing core areas.

## Supporting Information

**Appendix S1.** Genotyping and identification of unique sage-grouse individuals.

**Appendix S2.** Resistance surfaces derived from the distribution of seasonal habitat indices.

**Appendix S3.** Resistance surfaces derived from the distribution of landscape components.

**Figure S1.** Representation of transformation equations. **Figure S2.** Correlation between *G*_ST_ and Jost *D*_est_ for populations groupings. **Figure S3.** Strength of correlation among pairwise resistances with different transformations. **Figure S4.** Distribution and correlation among pairwise resistances derived from habitat suitability indices. **Figure S5.** Distribution and correlation among pairwise resistances derived from landscape components. **Figure S6.** Pairwise correlation for model selection criteria describing dispersal hypothesis between lek groupings of sage-grouse across Wyoming.

**Table S1.** Genetic summary statistics for sage-grouse lek groupings. **Table S2.** Univariate model selection criteria for habitat suitability indices describing functional connectivity. **Table S3.** Univariate model selection criteria for landscape components describing functional connectivity.
