# Supplementary material for: Landscape characteristics influencing the genetic structure of greater sage-grouse within the stronghold of their range: a holistic modeling approach
Source: Ecol Evol. 2015 May 1;5(10):1955–69. doi: 10.1002/ece3.1479 (PMC4449751; doi:10.1002/ece3.1479)
Supplement: Supplementary file 1 [file ece30005-1955-sd1.docx]

**Appendix S1:** **Genotyping and identification of unique sage-grouse individuals.**

DNA from 1,761 feather samples was extracted using a DNeasy Blood & Tissue Kit (Qiagen) following the user-developed protocol for feather samples. Portions of the extraction process were automated using a QiaCube (Qiagen). Samples were amplified across 14 grouse-specific microsatellite loci (MSP11, MSP18, SGMS06.6, SGMS06.8 (Oyler-McCance & St. John, 2010); SGCA5, SGCA11, SGCTAT1 (Taylor et al. 2003); TUT3 (Segelbacher et al. 2000); BG6 (Piertney and Höglund 2001); SG21, SG28, SG36, SG29, SG39; Fike et al. 2015). The primers for 2 loci were redesigned by T. Cross (pers. comm.) for better performance (SGCA5F: CGGACAGGTACATCCTGGAA, SGCA5R: GGGAAAAGATGTCAGAATCTACAAA, SGCA11F: GCAGTAAAGAAAATTTGGAAGCA, SGCA11R: TCTTGAACTGATGTTGGATTTG). Amplifications were performed in 10 L PCRs consisting of approximately 10 ng of template DNA, 0.2 mM of each dNTP, 0.25 M dye-labeled forward primer, 0.25 M reverse primer, 0.625 U GoTaq Flexi DNA polymerase (Promega), 2.25mM MgCl_2_ and 1X GoTaq Flexi Buffer (Promega). The amplification conditions for all loci were as follows: 94^o^C for 2 min, then 94^o^C for 1 min, annealing temperature (52 ^o^C : MSP11, SGMS06.8; 55 ^o^C : MSP18, SGCA5, BG6; 60 ^o^C : SGCA11, SGCTAT1, TUT3, SGMS06.6, SG21, SG28, SG36, SG29, SG39) for 1 min, and 72 ^o^C for 1 min for 40 cycles, then 60^o^C for 45 min and a final extension at 72^o^C for 5 min. The sex of each sample was determined by amplifying a region of the CDH gene using the primers 1237L and 1272H (Kahn et al. 1998). Amplification of the sexing marker was the same as for the microsatellites with the exception of a 56^o^C annealing temperature. PCR products were multi-loaded based on product size and primer label, combined with GeneScan LIZ 600 internal lane size standard (Applied Biosystems), and electrophoresed through a capillary gel matrix using an AB3500 Automated DNA Sequencer (Applied Biosystems). Allele sizes were determined for each locus using GeneMapper v4.1 software (Applied Biosystems). After the first round of genotyping, poor quality samples (that amplified at < 7 loci) and those that were flagged as duplicates by program DROPOUT (McKelvey and Schwartz 2005) were removed. In Dropout we removed all duplicate individuals whose genotypes differed from a sample that was retained at less than 3 loci, including missing data. The remaining 1056 samples were then genotyped at least one more time to verify the genotype and identify instances of genotyping error and allelic dropout.

As we are using a non-invasive sample-collection method, the true identity of each sample was unknown introducing the possibility of repeated sampling. Therefore, we then again identified and eliminated any repeated samples, while accounting for genotyping error and missing data by combining two approaches. First, using the program DROPOUT (McKelvey and Schwartz 2005) we matched all individuals whose genotype differed at less than 3 loci, regardless of the amount of missing data. We regarded this estimate as conservative, as some relatedness within leks was expected (Bush et al. 2010), and differing by 3 loci could be significant for some individuals with missing data. Thus, we compared the DROPOUT results with matches identified using ALLELEMATCH (Galpern et al. 2012). ALLELEMATCH accounts for missing data, but requires a cutoff threshold, which is roughly equivalent to number of mismatched alleles between samples. We determined the cutoff off threshold using an optimization procedure (*amUniqueProfile*) within the program.

DROPOUT indicated 117 pairs of samples matching at 3 or fewer loci. Running *amUniqueProfile* in ALLELEMATCH the ideal alleleMismatch parameter suggested was 2. Some preliminary comparisons with DROPOUT, however, suggested this value was likely too low and we increased the alleleMatch parameter to 4, which resulted in 27 fewer matches than DROPOUT, but no additional matches. To derive a final dataset of unique individuals we first removed the individuals with an exact match or an exact match except for missing data. In all cases we merged the genotypes to have the lowest amount of missing data. For the non-exact matches grouped by both ALLELEMATCH and DROPOUT we used the following rules to derive a unique genotype, 1) if samples differed by homozygous/heterozygous data we called the heterozygote as the true genotype, 2) if there were different alleles altogether we coded the locus as missing data. Lastly, we examined the 27 matches unique to DROPOUT that were not identified as matches in ALLELEMATCH. In most cases, the differences were due to a combination of missing data (i.e., data are missing for a locus in one genotype, but not the other) and real allelic differences. Because ALLELEMATCH has a penalty for missing data these were not considered matches. Eighteen of the 27 cases were heterozygote/homozygote mismatches and thus, these were merged as the same individual, keeping the heterozygous locus data. Nine of the 27 had at least one allele with different data (not simply a heterozygote/homozygote mismatch) and these were classed as different individuals, with both individuals retained.

**References**

Bush, K. L., C. L. Aldridge, J. E. Carpenter, C. A. Paszkowski, M. S. Boyce, and D. W. Coltman. 2010. Birds of a feather do not always lek together: genetic diversity and kinship structure of greater sage-grouse (*Centrocercus urophasianus*) in Alberta 127:343–353.

Fike J. A., S. J. Oyler-McCance, S. J. Zimmerman, and T.A. Castoe. 2015. Development of 13 microsatellites for Gunnison Sage-grouse (*Centrocercus minimus*) using next-generation shotgun sequencing and their utility in Greater Sage-grouse (*Centrocercus urophasianus*). Conserv. Genet. Resour. **7**:211-214.

Galpern, P., M. Manseau, P. Hettinga, K. Smith, and P. Wilson. 2012. Allelematch: an R package for identifying unique multilocus genotypes where genotyping error and missing data may be present. Mol. Ecol. Resour. 12:771–778.

Kahn, N. W., J. St. John, and T. W. Quinn. 1998. Chromosome-specific intron size differences in the avian CHD gene provide an efficient method for sex identification in birds. Auk:1074–1078.

McKelvey, K. S., and M. K. Schwartz. 2005. DROPOUT: a program to identify problem loci and samples for noninvasive genetic samples in a capture-mark-recapture framework. Mol. Ecol. Notes 5:716–718.

Oyler-McCance, S. J., and J. St. John. 2010. Characterization of small microsatellite loci for use in non invasive sampling studies of Gunnison Sage-grouse (*Centrocercus minimus*). Conserv. Genet. Resour. 2:17–20.

Piertney, S., and J. Höglund. 2001. Polymorphic microsatellite DNA markers in black grouse (*Tetrao tetrix*). Mol. Ecol. Notes 1:303–304.

Segelbacher, G., R. J. Paxton, G. Steinbrück, P. Trontelj, and I. Storch. 2000. Characterization of microsatellites in capercaillie Tetrao urogallus (AVES). Mol. Ecol. 9:1934–1935.

Taylor, S., S. Oyler-McCance, and T. Quinn. 2003. Isolation and characterization of microsatellite loci in Greater Sage-Grouse (*Centrocercus urophasianus*). Mol. Ecol. Notes 3:262–264.
