# Supplementary material for: Landscape characteristics influencing the genetic structure of greater sage-grouse within the stronghold of their range: a holistic modeling approach
Source: Ecol Evol. 2015 May 1;5(10):1955–69. doi: 10.1002/ece3.1479 (PMC4449751; doi:10.1002/ece3.1479)
Supplement: Supplementary file 2 [file ece30005-1955-sd2.pdf]

Appendix S2. Resistance surfaces describing functional connectivity for sage-grouse across Wyoming. Surfaces were derived from the distribution of seasonal habitat indices and transformed using a moving window and exponential equations.

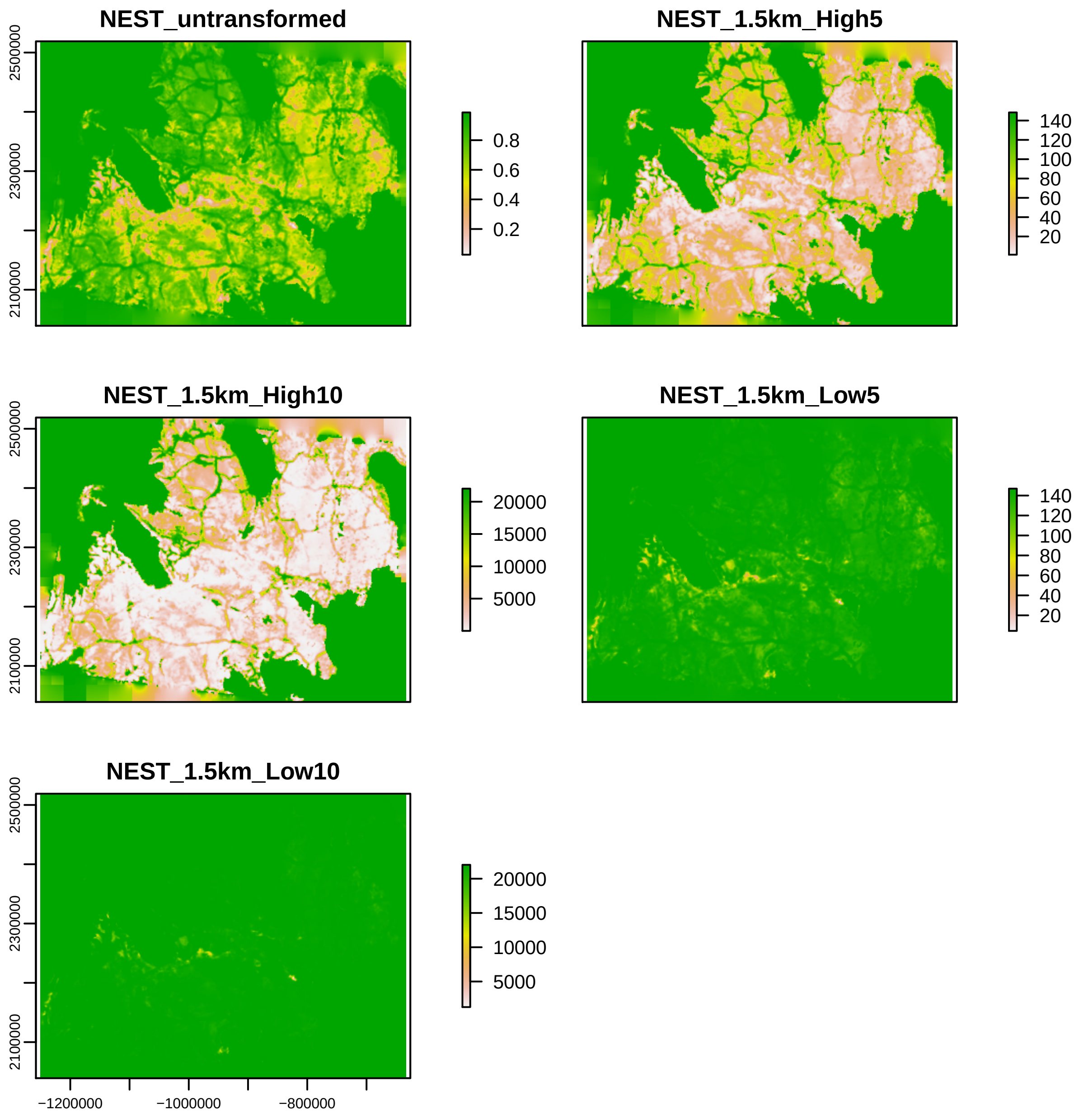

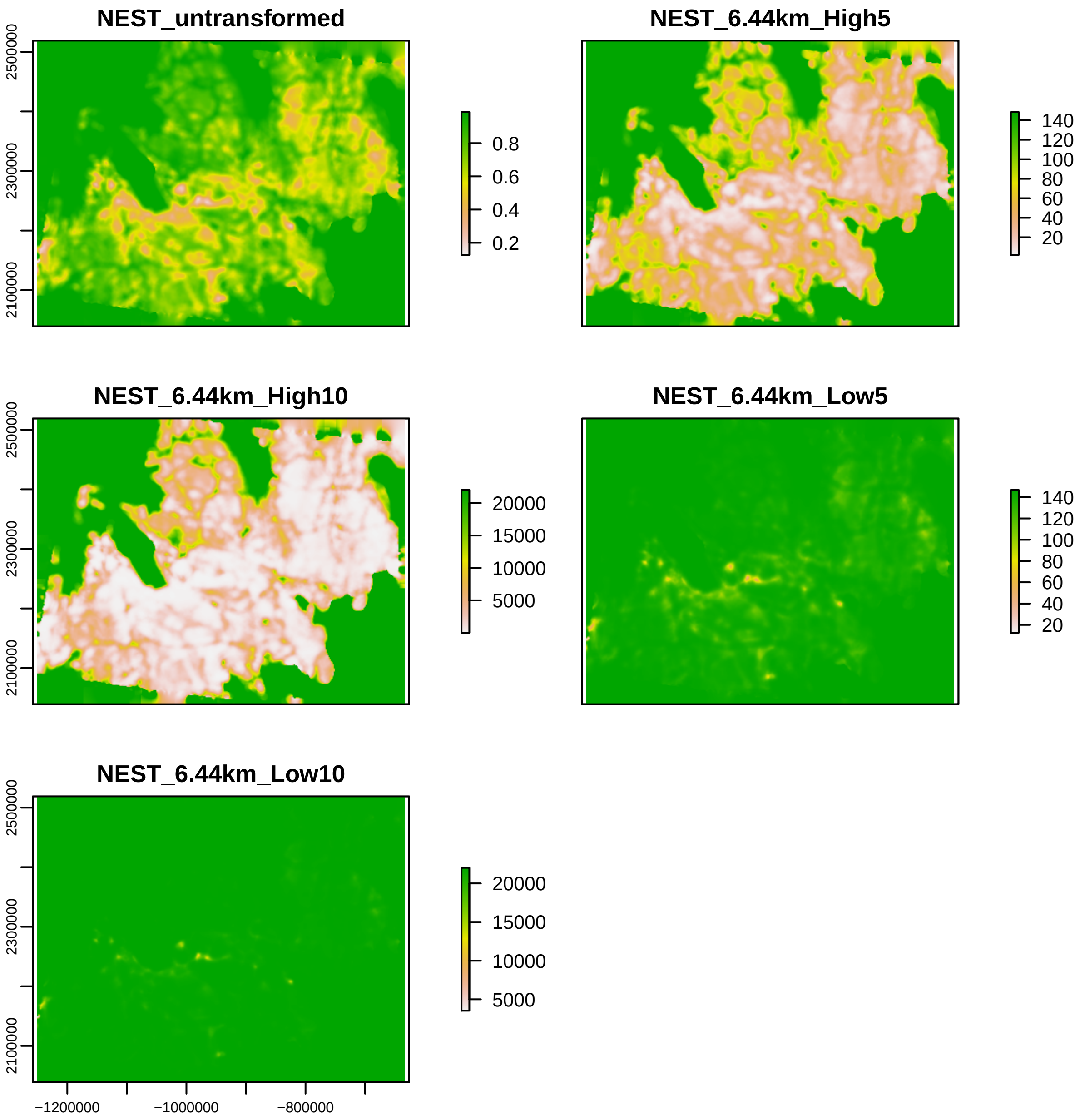
NEST_untransformed
NEST_6.44km_High5
NEST_6.44km_High10
NEST_6.44km_Low5
NEST_6.44km_Low10
2500000
2300000
2100000
−1200000
−1000000
−800000
0.8
0.6
0.4
0.2
140
120
100
80
60
40
20
20000
15000
10000
5000

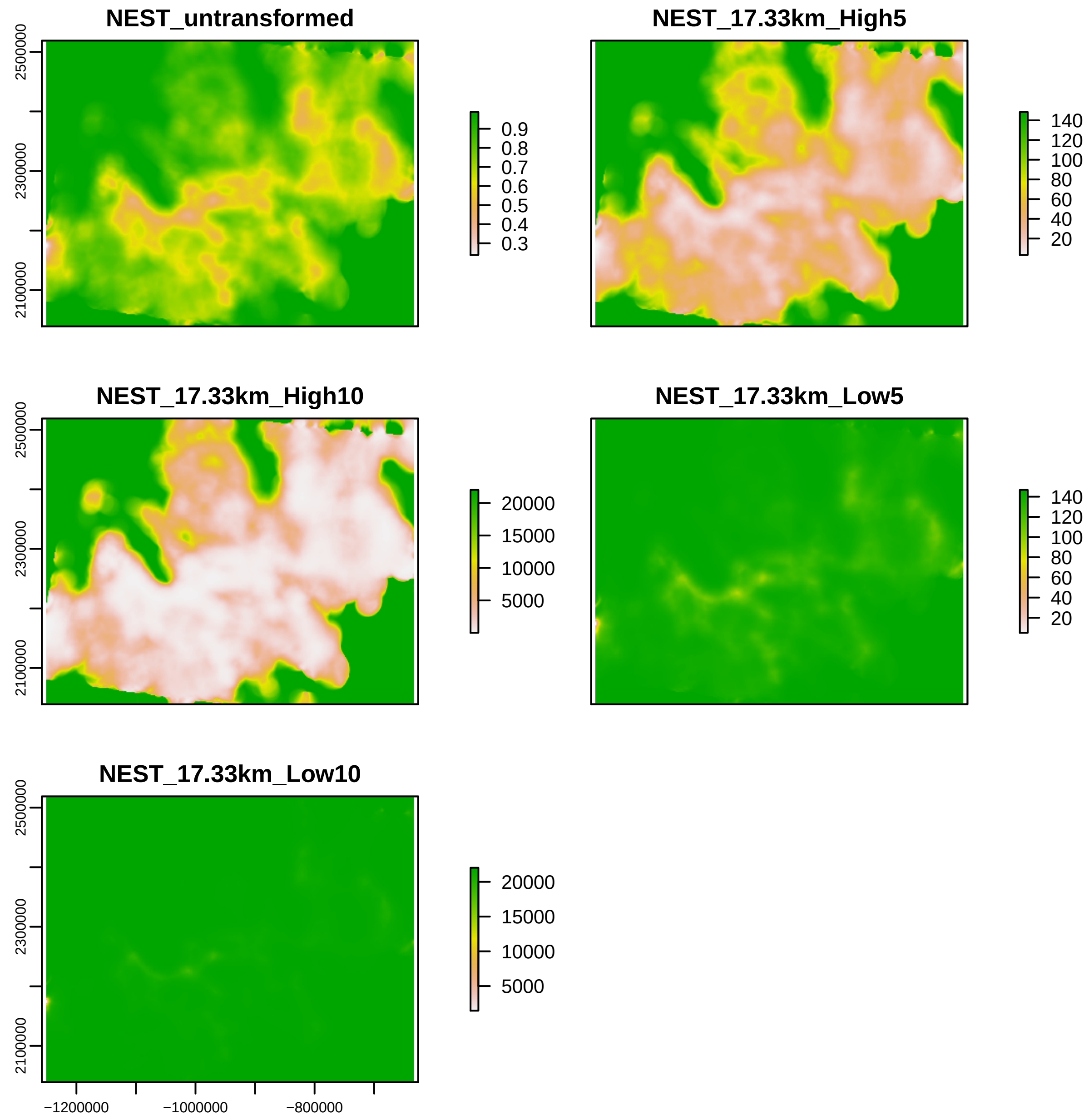
NEST_untransformed
NEST_17.33km_High5
NEST_17.33km_High10
NEST_17.33km_Low5
NEST_17.33km_Low10
2500000
2300000
2100000
0.9
0.8
0.7
0.6
0.5
0.4
0.3
140
120
100
80
60
40
20
20000
15000
10000
5000
−1200000
−1000000
−800000

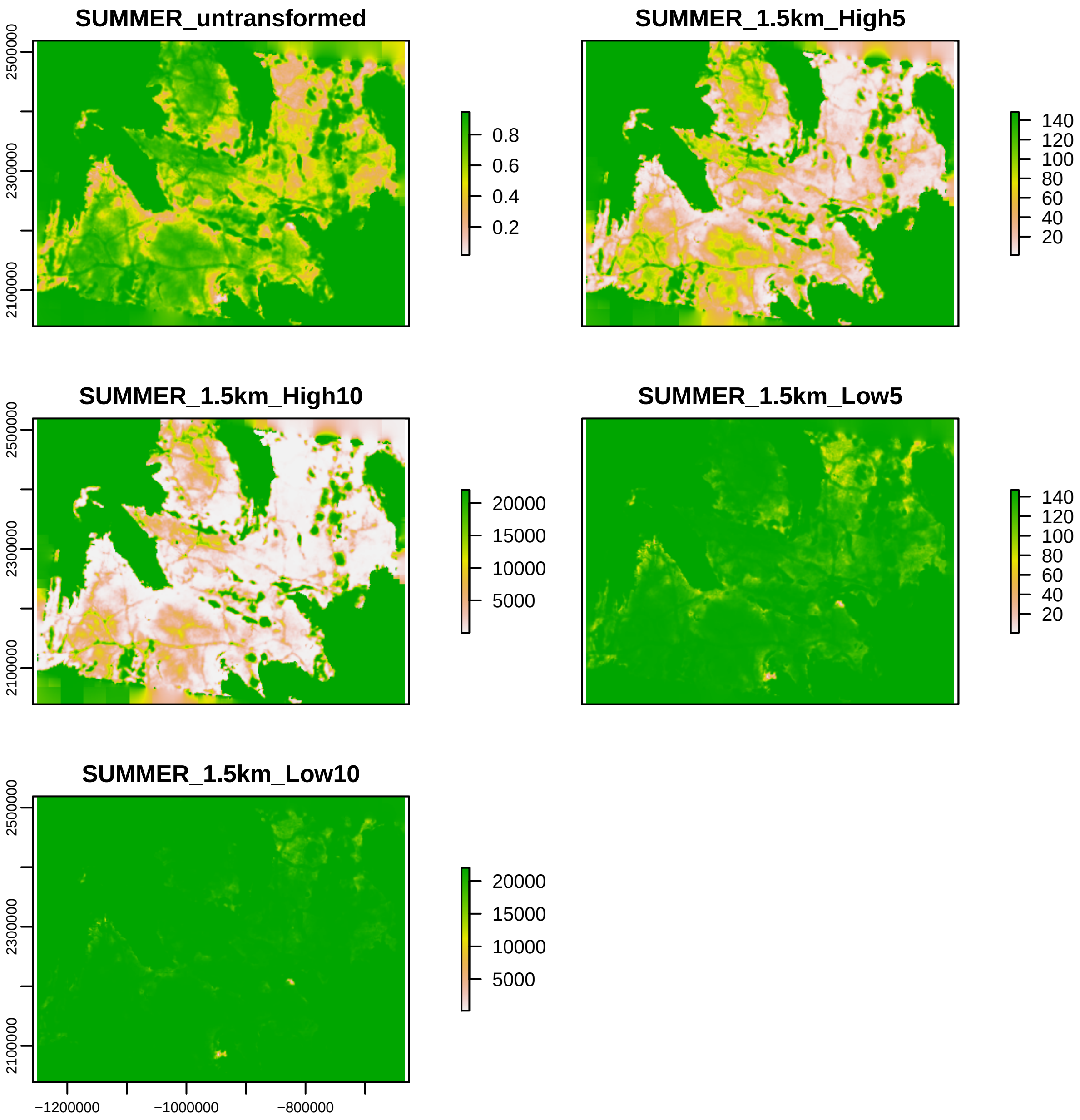
SUMMER_untransformed
SUMMER_1.5km_High5
SUMMER_1.5km_High10
SUMMER_1.5km_Low5
SUMMER_1.5km_Low10
2500000
2300000
2100000
−1200000
−1000000
−800000
0.8
0.6
0.4
0.2
140
120
100
80
60
40
20
20000
15000
10000
5000

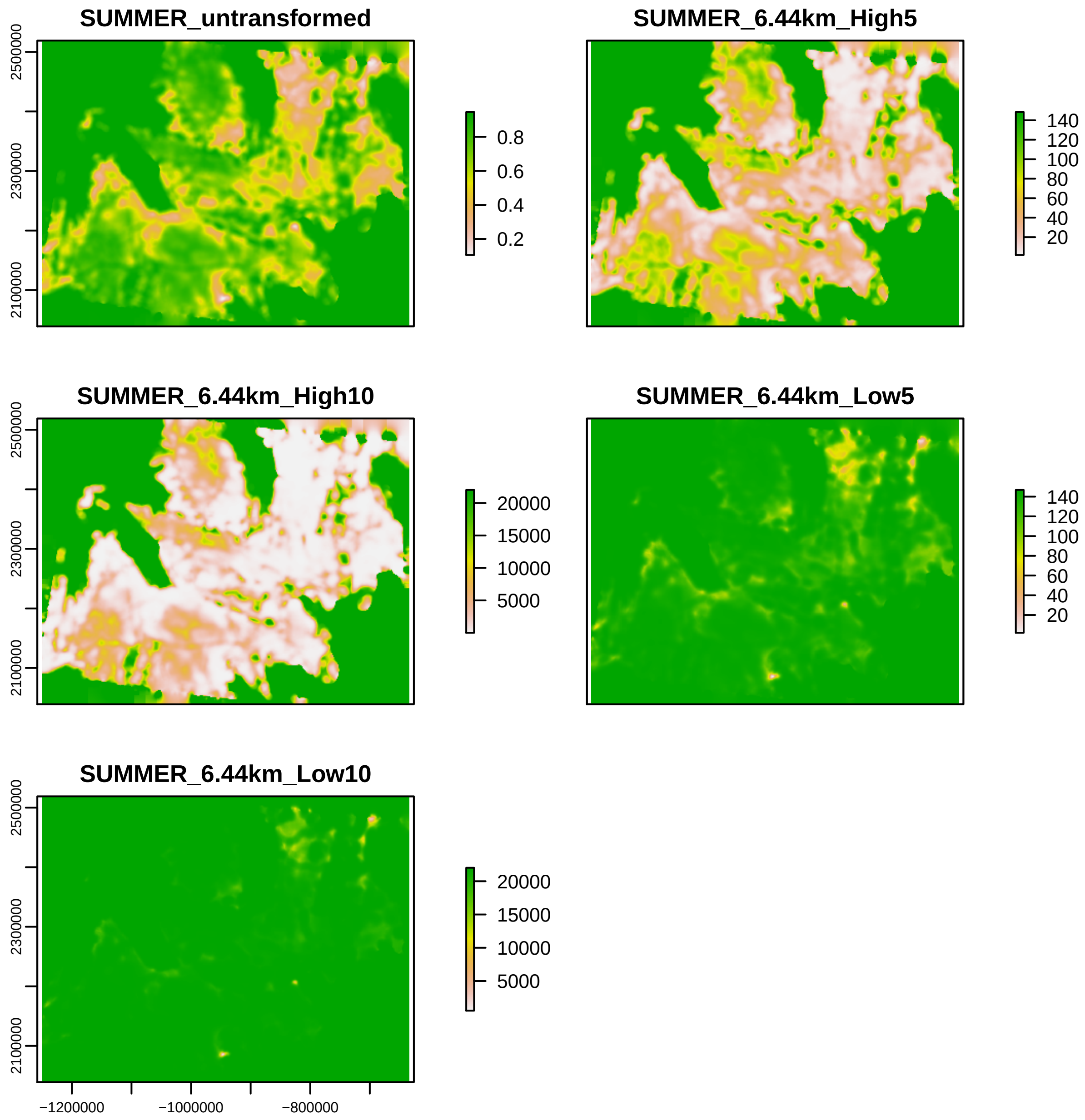
SUMMER_untransformed
SUMMER_6.44km_High5
SUMMER_6.44km_High10
SUMMER_6.44km_Low5
SUMMER_6.44km_Low10
2500000
2300000
2100000
−1200000
−1000000
−800000
0.8
0.6
0.4
0.2
140
120
100
80
60
40
20
20000
15000
10000
5000

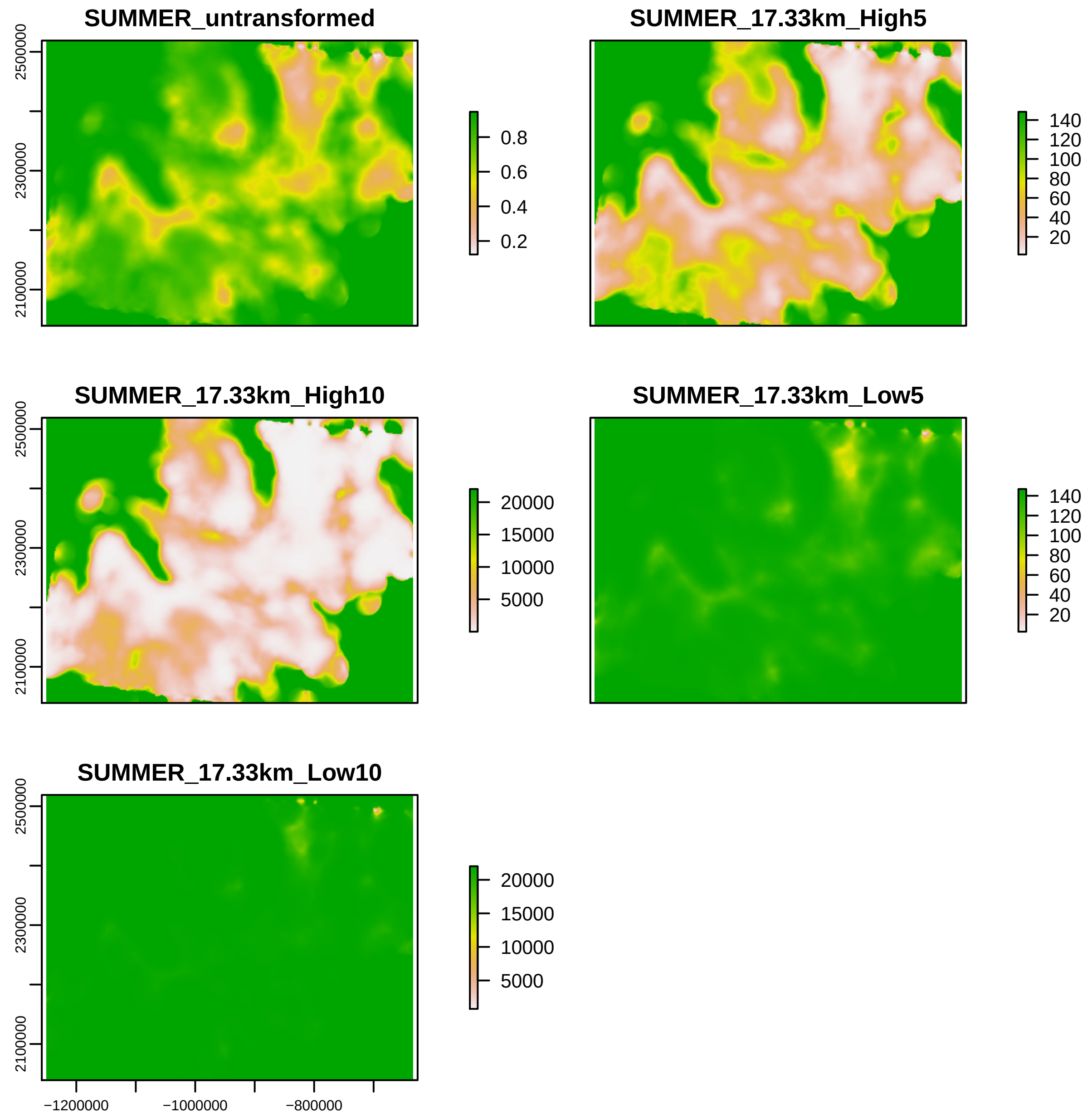
SUMMER_untransformed
SUMMER_17.33km_High5
SUMMER_17.33km_High10
SUMMER_17.33km_Low5
SUMMER_17.33km_Low10
2500000
2300000
2100000
−1200000
−1000000
−800000
0.8
0.6
0.4
0.2
140
120
100
80
60
40
20
20000
15000
10000
5000

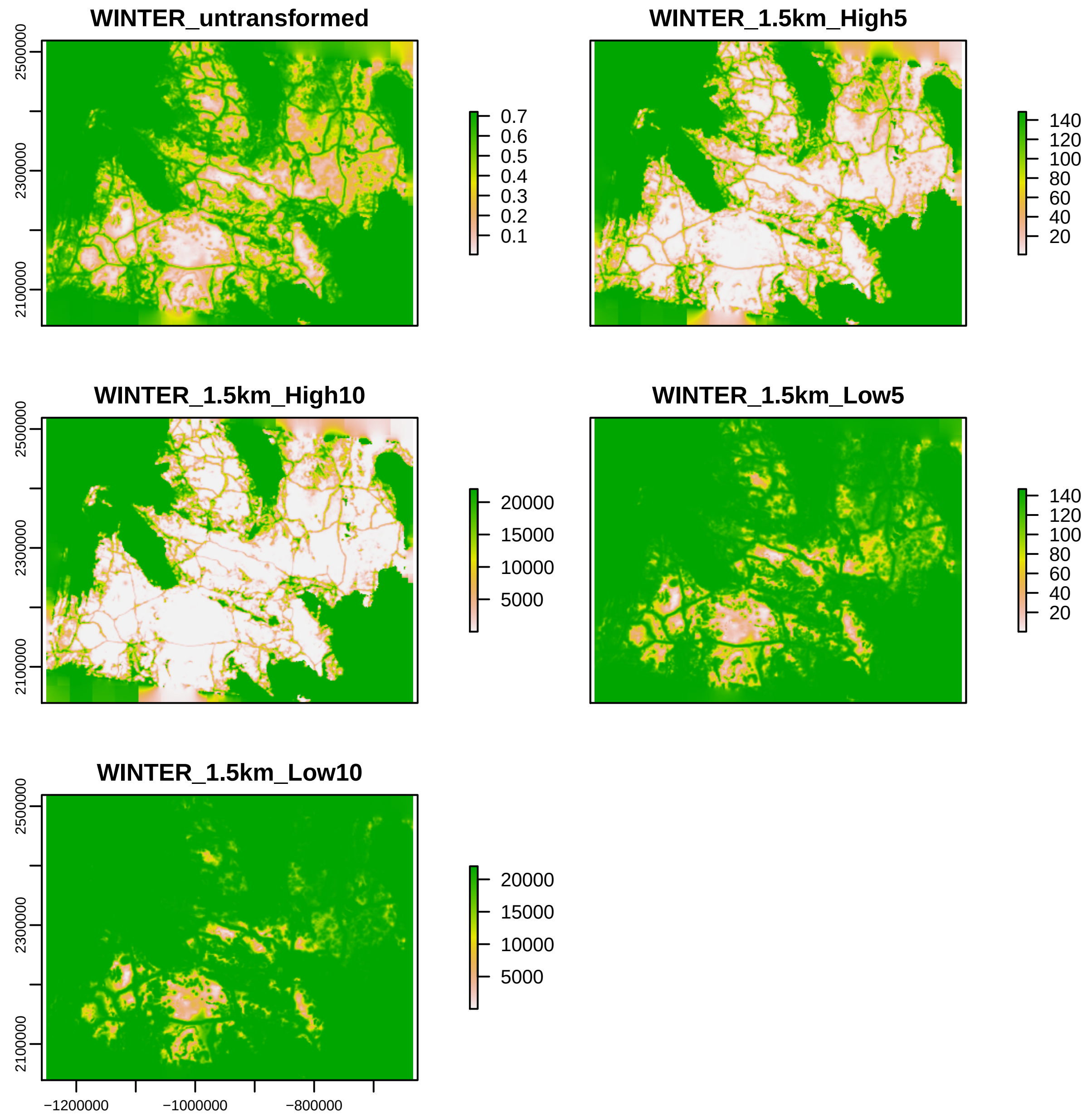
WINTER_untransformed
WINTER_1.5km_High5
WINTER_1.5km_High10
WINTER_1.5km_Low5
WINTER_1.5km_Low10
2500000
2300000
2100000
−1200000
−1000000
−800000
0.7
0.6
0.5
0.4
0.3
0.2
0.1
140
120
100
80
60
40
20
20000
15000
10000
5000

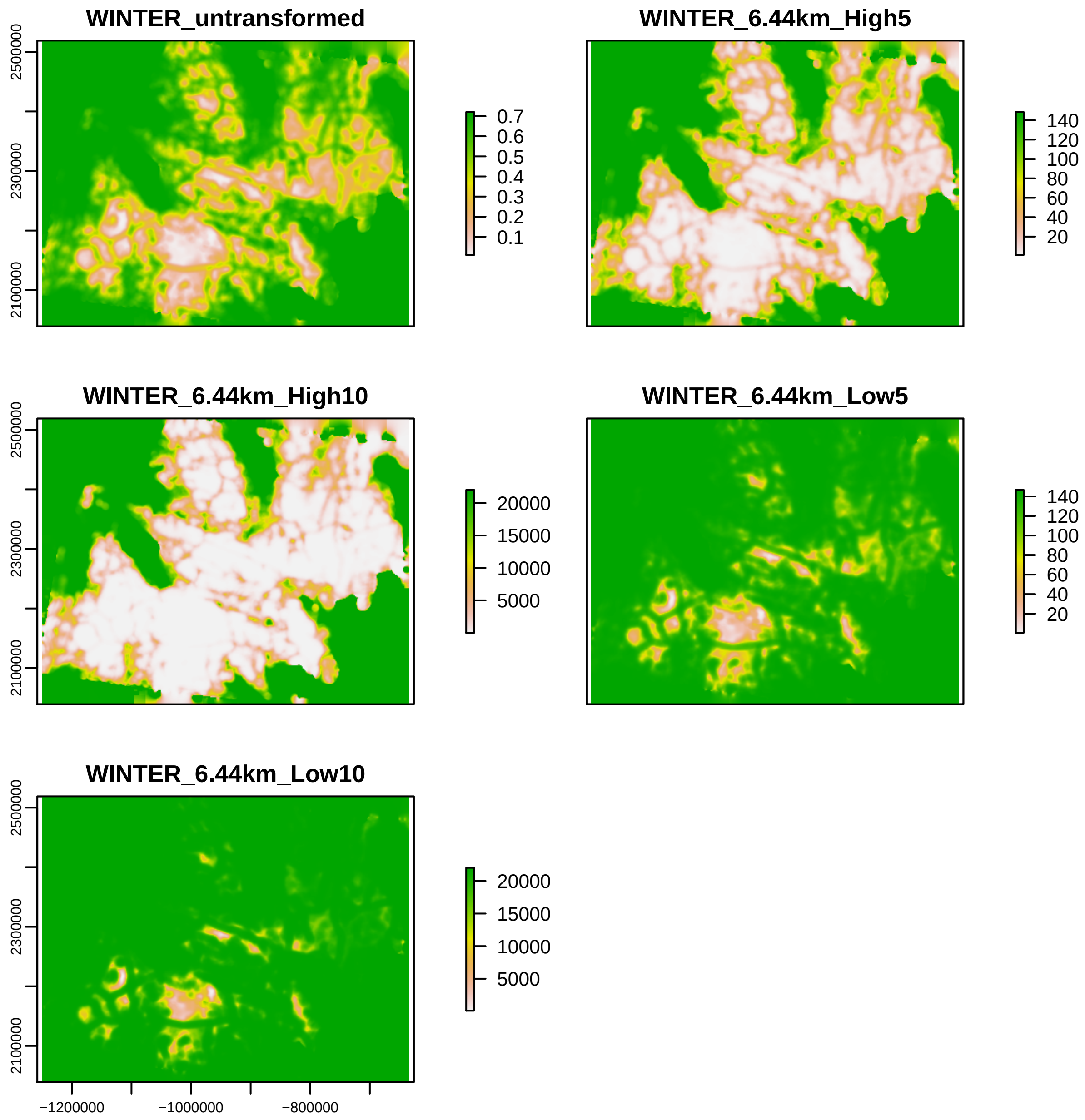
WINTER_untransformed
WINTER_6.44km_High5
WINTER_6.44km_High10
WINTER_6.44km_Low5
WINTER_6.44km_Low10
2500000
2300000
2100000
−1200000
−1000000
−800000
0.7
0.6
0.5
0.4
0.3
0.2
0.1
140
120
100
80
60
40
20
20000
15000
10000
5000

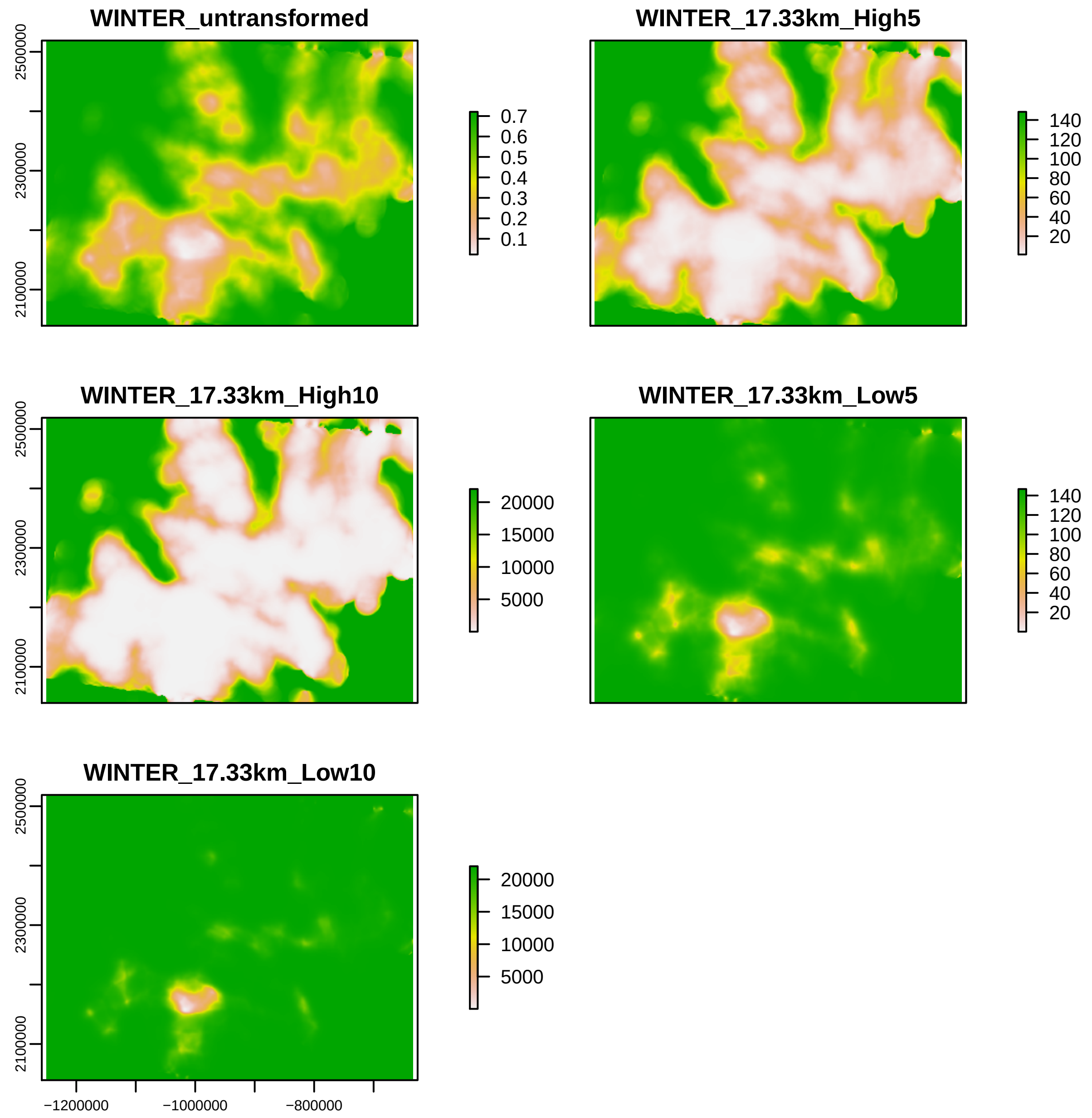
WINTER_untransformed
WINTER_17.33km_High5
WINTER_17.33km_High10
WINTER_17.33km_Low5
WINTER_17.33km_Low10
2500000
2300000
2100000
−1200000
−1000000
−800000
0.7
0.6
0.5
0.4
0.3
0.2
0.1
140
120
100
80
60
40
20
20000
15000
10000
5000
