# Supplementary material for: Landscape characteristics influencing the genetic structure of greater sage-grouse within the stronghold of their range: a holistic modeling approach
Source: Ecol Evol. 2015 May 1;5(10):1955–69. doi: 10.1002/ece3.1479 (PMC4449751; doi:10.1002/ece3.1479)
Supplement: Supplementary file 3 [file ece30005-1955-sd3.pdf]

Appendix S3. Resistance surfaces describing functional connectivity for sage-grouse across Wyoming. Surfaces were derived from the distribution of landscape components and transformed using moving windows and exponential equations.

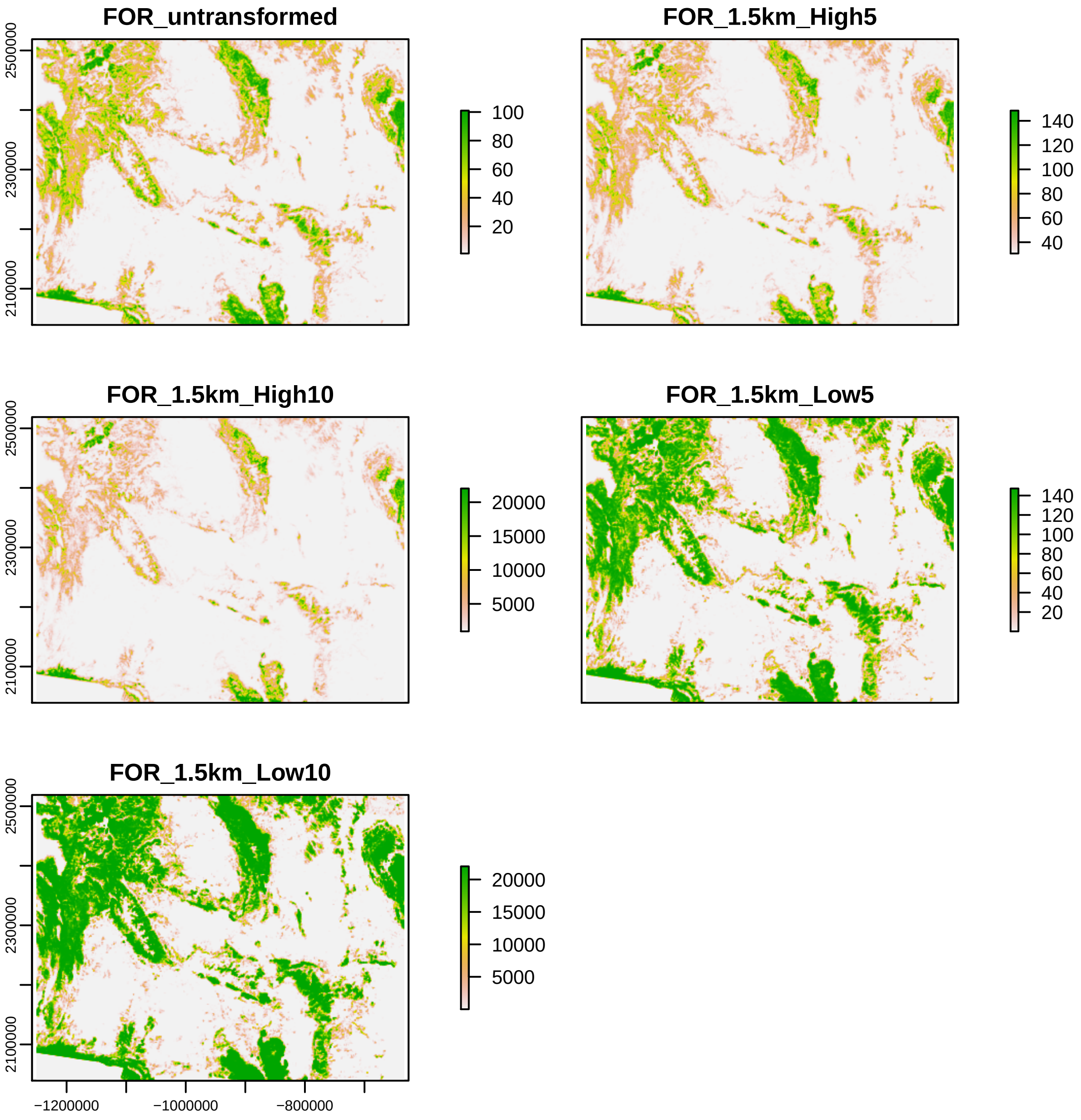

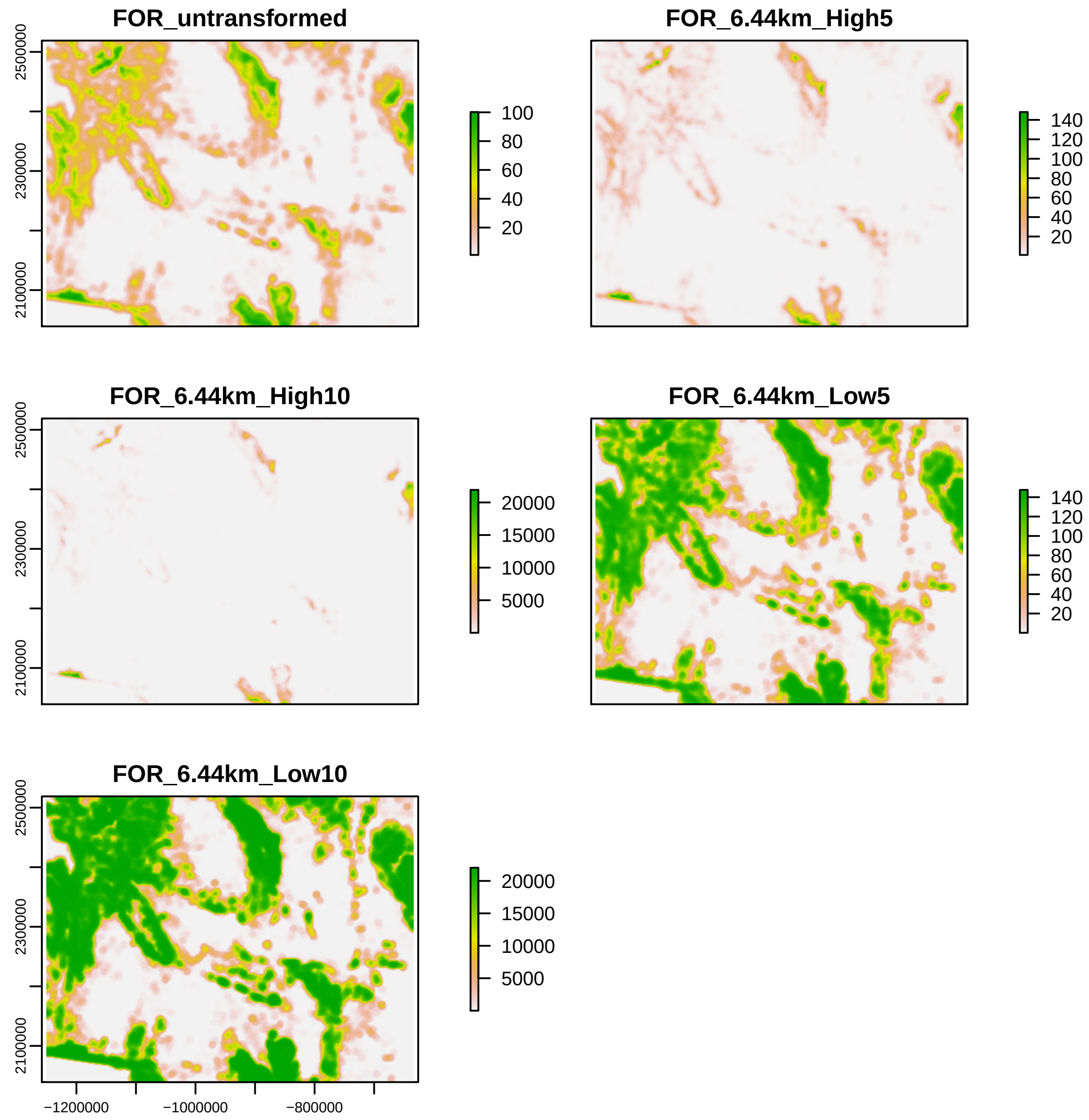
FOR_untransformed
FOR_6.44km_High5
FOR_6.44km_High10
FOR_6.44km_Low5
FOR_6.44km_Low10
2500000
2300000
2100000
−1200000
−1000000
−800000
100
80
60
40
20
140
120
100
80
60
40
20
20000
15000
10000
5000

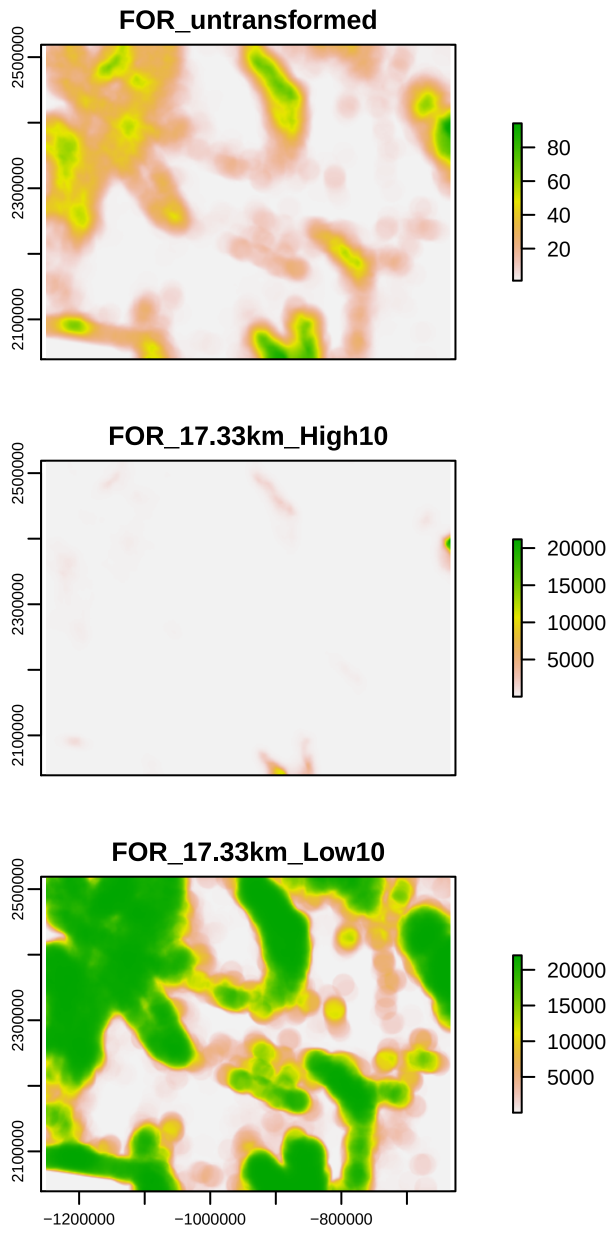
FOR_untransformed
FOR_17.33km_High5
FOR_17.33km_High10
FOR_17.33km_Low5
FOR_17.33km_Low10
2500000
2300000
2100000
−1200000
−1000000
−800000
80
60
40
20
140
120
100
20000
15000
10000
5000

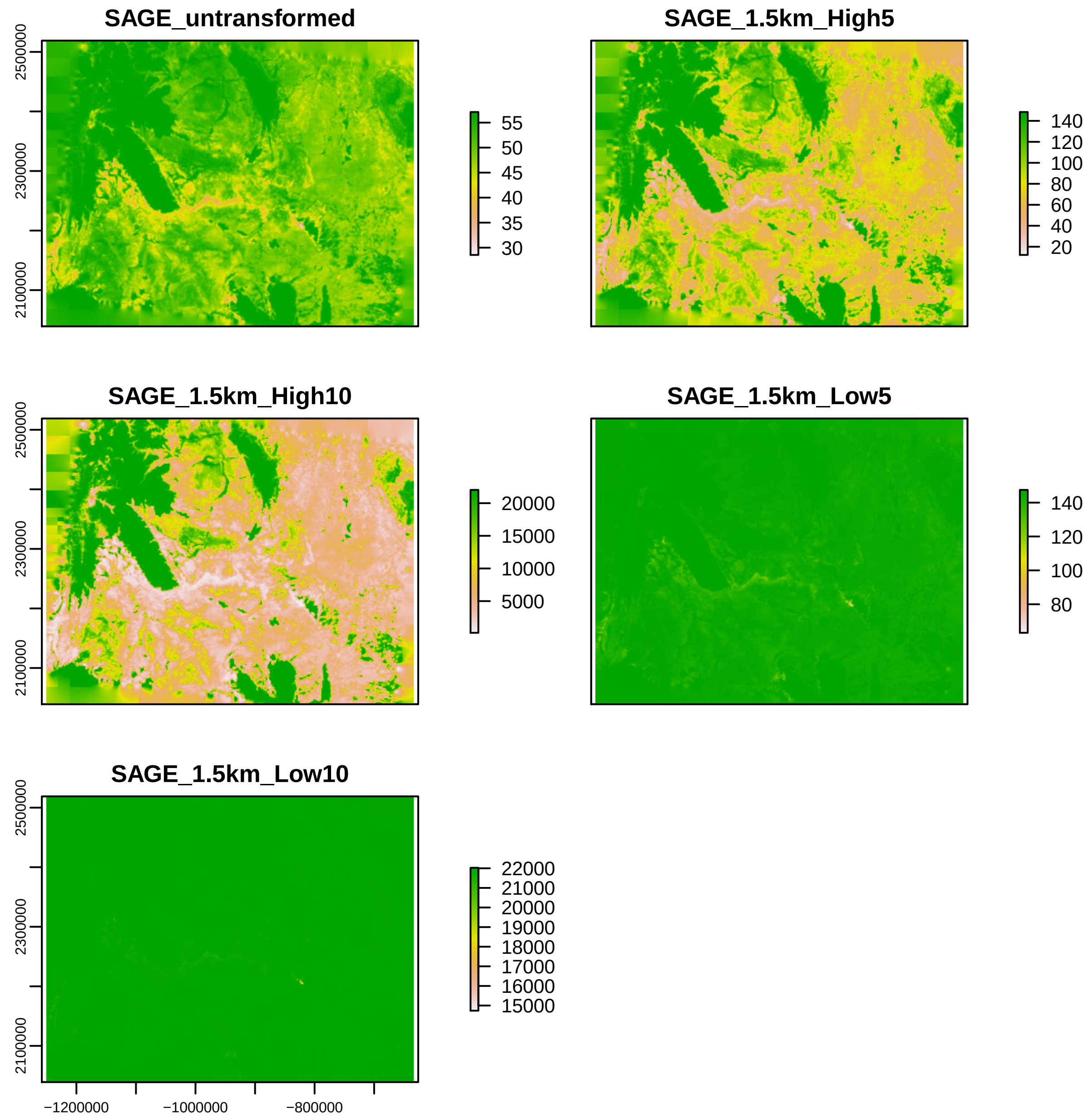
SAGE_untransformed
SAGE_1.5km_High5
SAGE_1.5km_High10
SAGE_1.5km_Low5
SAGE_1.5km_Low10
2500000
2300000
2100000
−1200000
−1000000
−800000
55
50
45
40
35
30
140
120
100
80
60
40
20
20000
15000
10000
5000
140
120
100
80
22000
21000
20000
19000
18000
17000
16000
15000

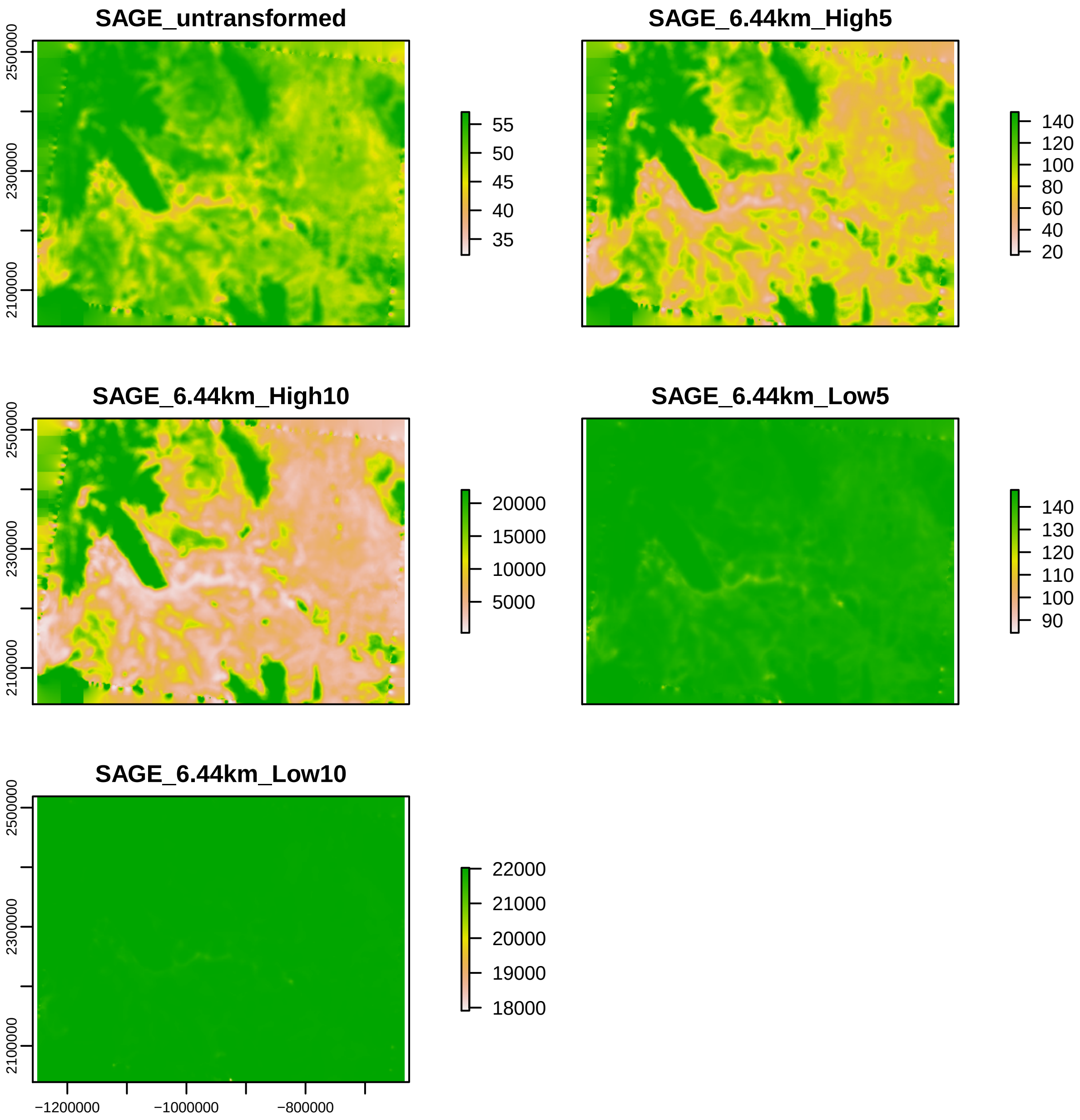
SAGE_untransformed
SAGE_6.44km_High5
SAGE_6.44km_High10
SAGE_6.44km_Low5
SAGE_6.44km_Low10
2500000
2300000
2100000
−1200000
−1000000
−800000
55
50
45
40
35
140
120
100
80
60
40
20
20000
15000
10000
5000
140
130
120
110
100
90
22000
21000
20000
19000
18000

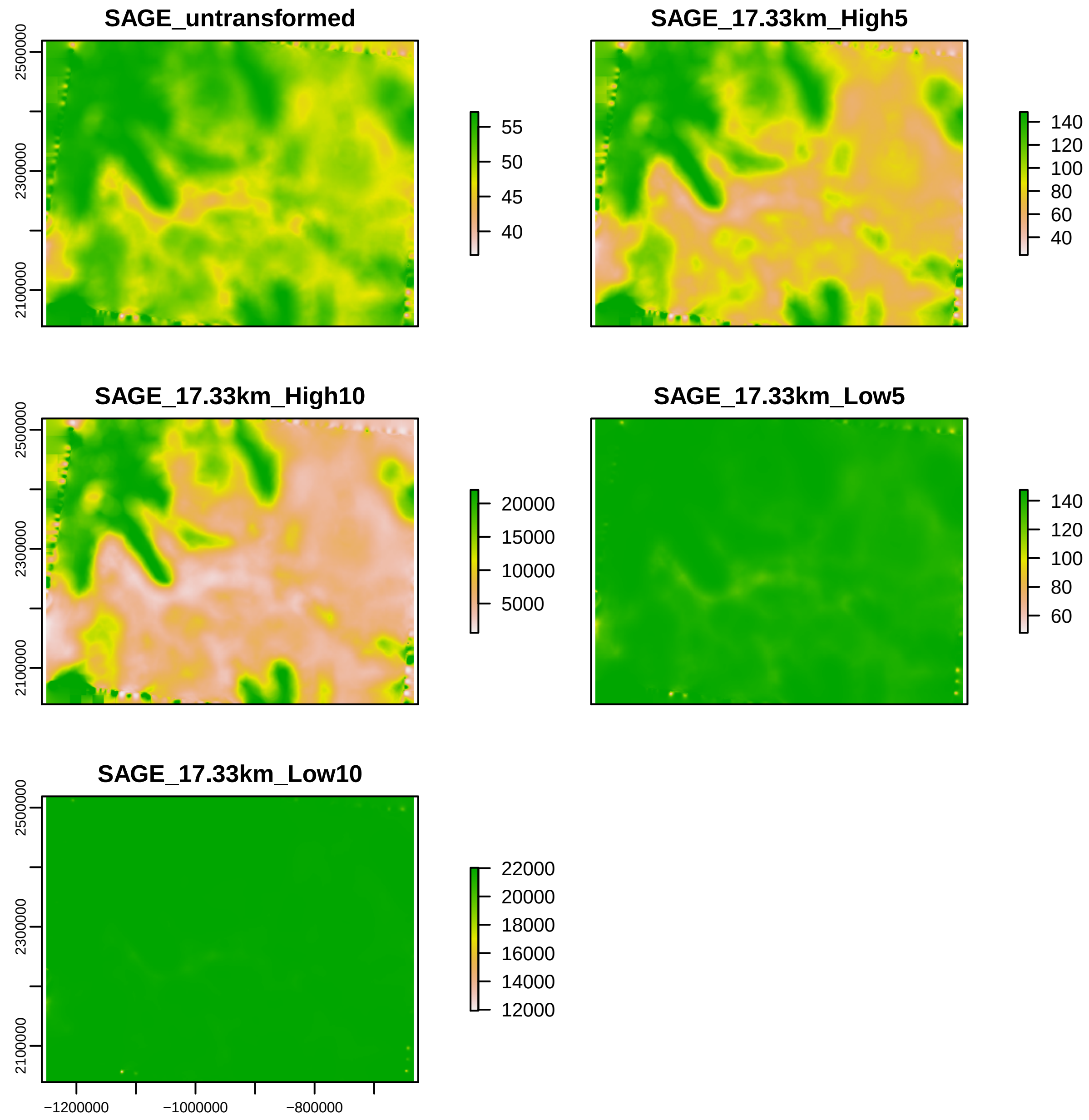
SAGE_untransformed
SAGE_17.33km_High5
SAGE_17.33km_High10
SAGE_17.33km_Low5
SAGE_17.33km_Low10
2500000
2300000
2100000
−1200000
−1000000
−800000
55
50
45
40
140
120
100
80
60
40
20000
15000
10000
5000
140
120
100
80
60
22000
20000
18000
16000
14000
12000

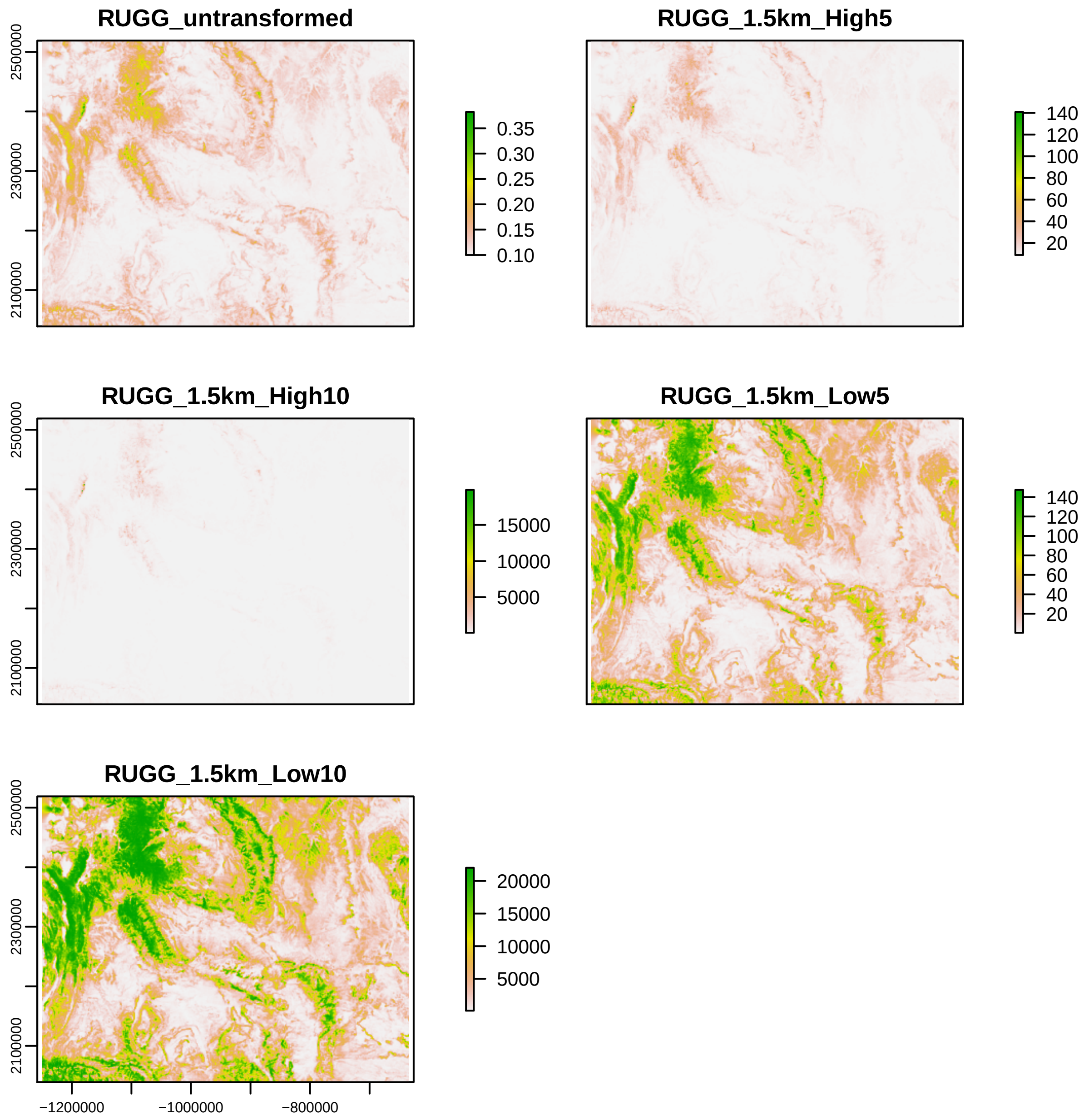
RUGG_untransformed
RUGG_1.5km_High5
RUGG_1.5km_High10
RUGG_1.5km_Low5
RUGG_1.5km_Low10
2500000
2300000
2100000
0.35
0.30
0.25
0.20
0.15
0.10
140
120
100
80
60
40
20
15000
10000
5000
20000
−1200000
−1000000
−800000

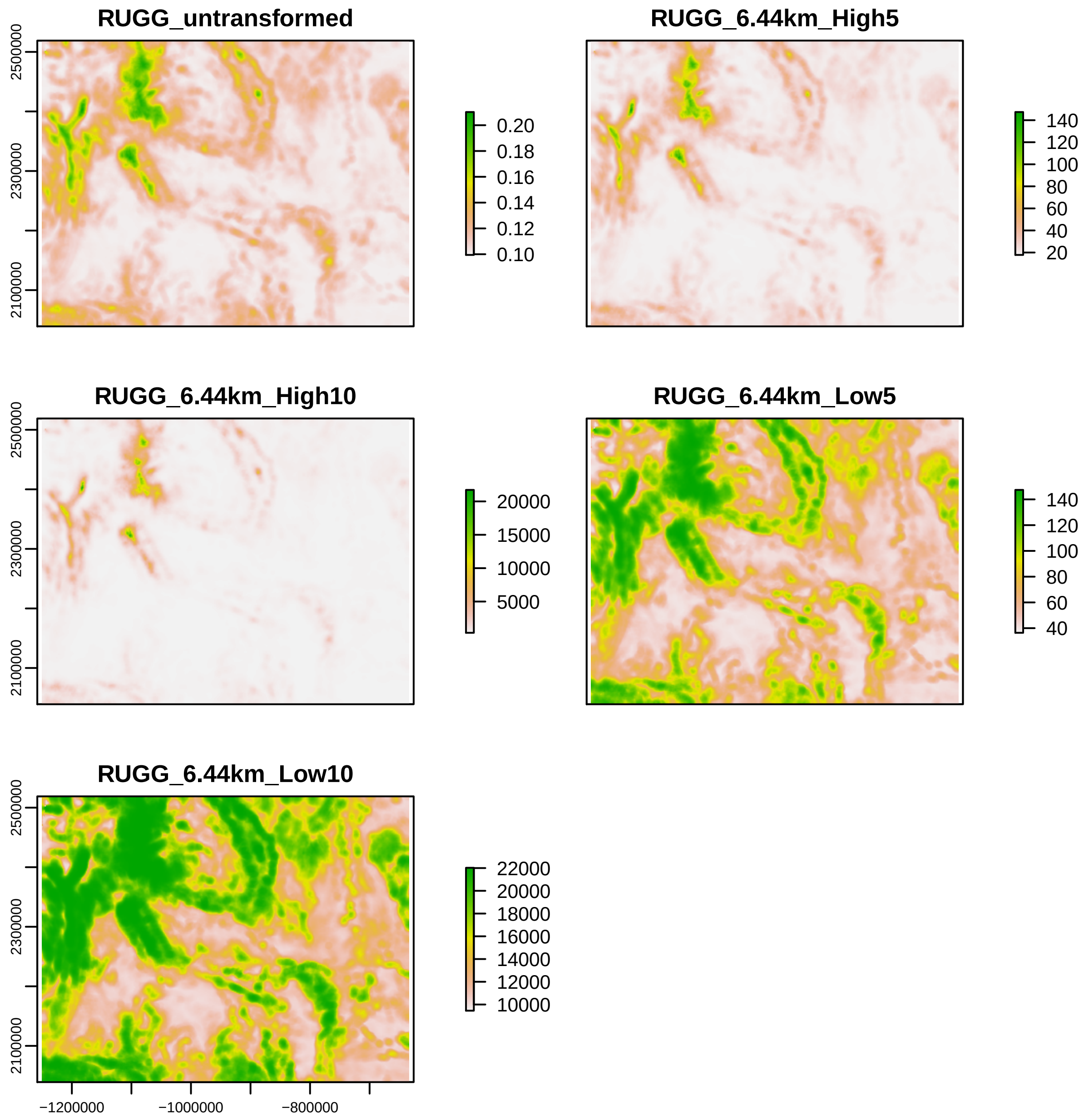
RUGG_untransformed
RUGG_6.44km_High5
RUGG_6.44km_High10
RUGG_6.44km_Low5
RUGG_6.44km_Low10
2500000
2300000
2100000
−1200000
−1000000
−800000
0.20
0.18
0.16
0.14
0.12
0.10
140
120
100
80
60
40
20
20000
15000
10000
5000
22000
18000
16000
14000
12000

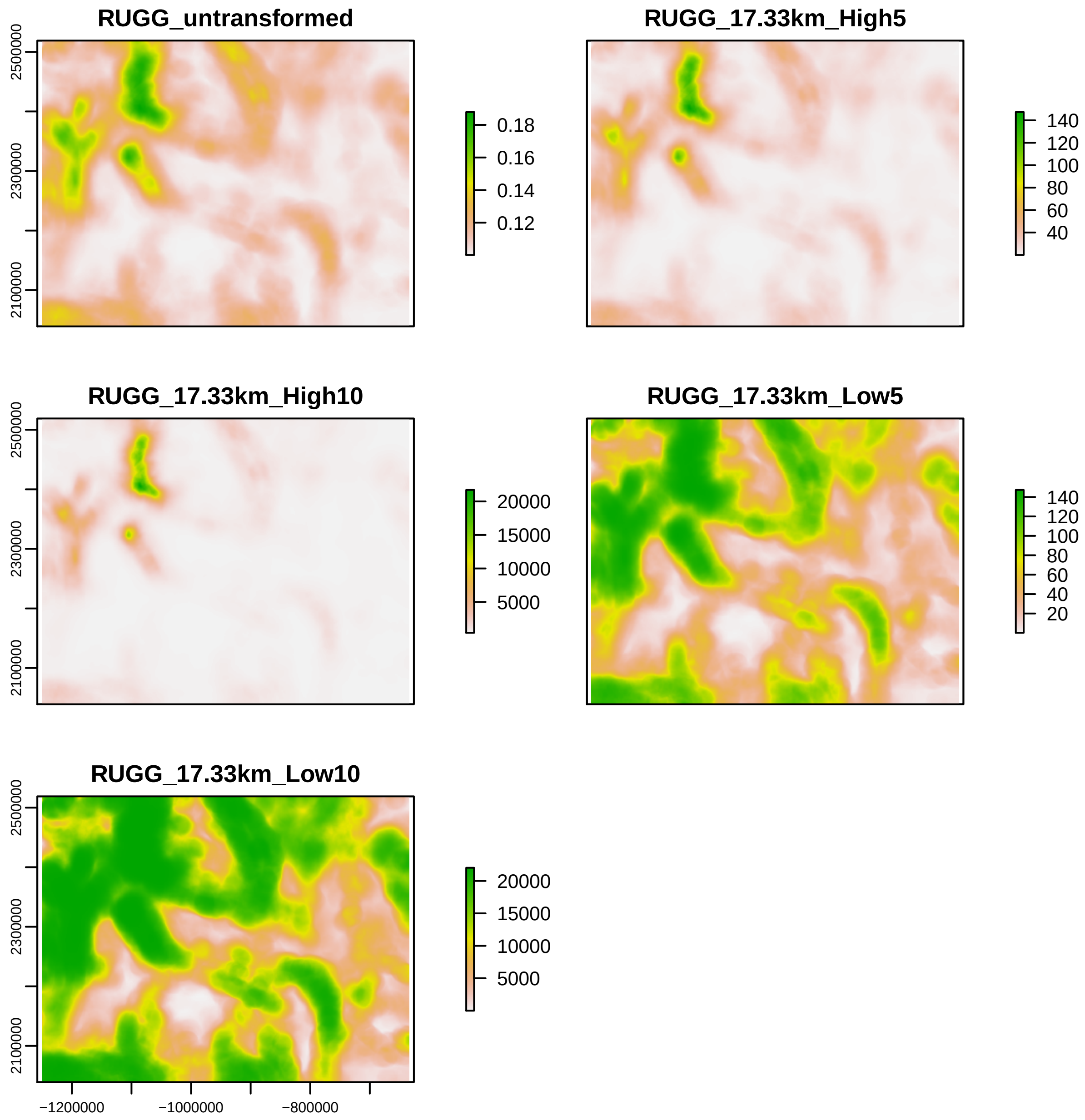
RUGG_untransformed
RUGG_17.33km_High5
RUGG_17.33km_High10
RUGG_17.33km_Low5
RUGG_17.33km_Low10
2500000
2300000
2100000
−1200000
−1000000
−800000
0.18
0.16
0.14
0.12
140
120
100
80
60
40
20
20000
15000
10000
5000

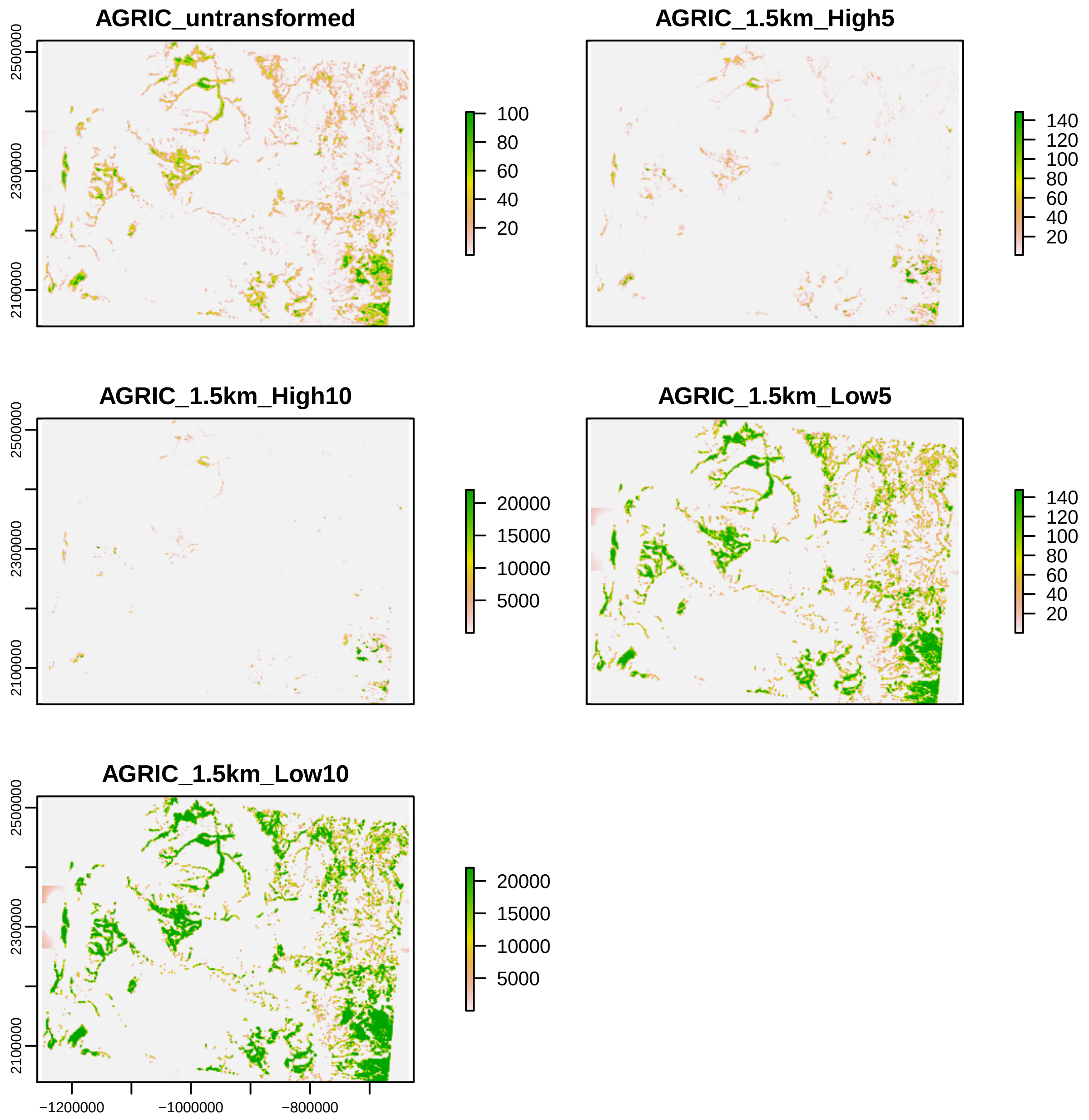
AGRIC_untransformed
AGRIC_1.5km_High5
AGRIC_1.5km_High10
AGRIC_1.5km_Low5
AGRIC_1.5km_Low10
2500000
2300000
2100000
−1200000
−1000000
−800000
100
80
60
40
20
140
120
100
80
60
40
20
20000
15000
10000
5000

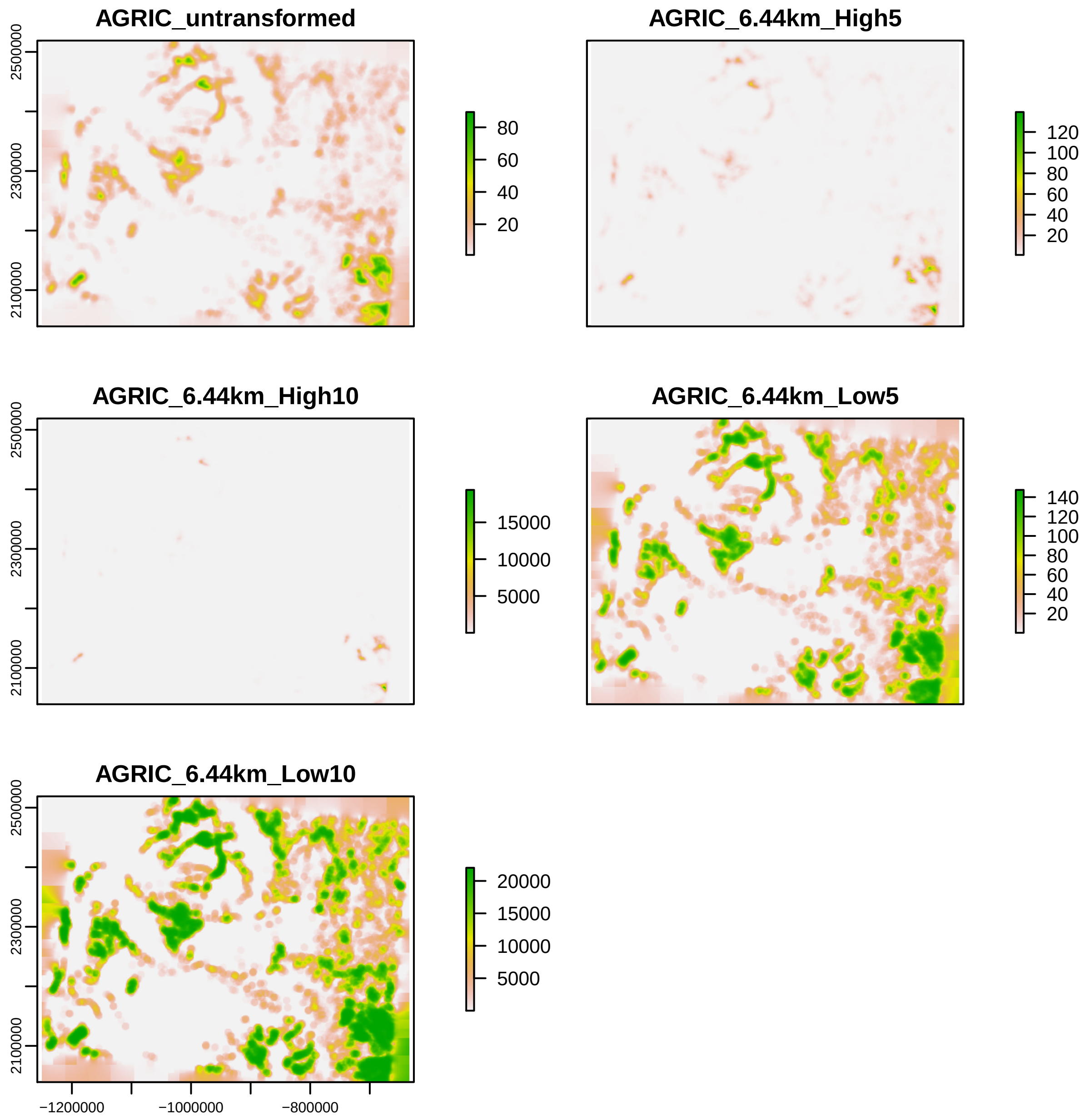
AGRIC_untransformed
AGRIC_6.44km_High5
AGRIC_6.44km_High10
AGRIC_6.44km_Low5
AGRIC_6.44km_Low10
2500000
2300000
2100000
−1200000
−1000000
−800000
80
60
40
20
120
100
80
60
40
20
15000
10000
5000
140
120
100
80
60
40
20
20000
15000
10000
5000

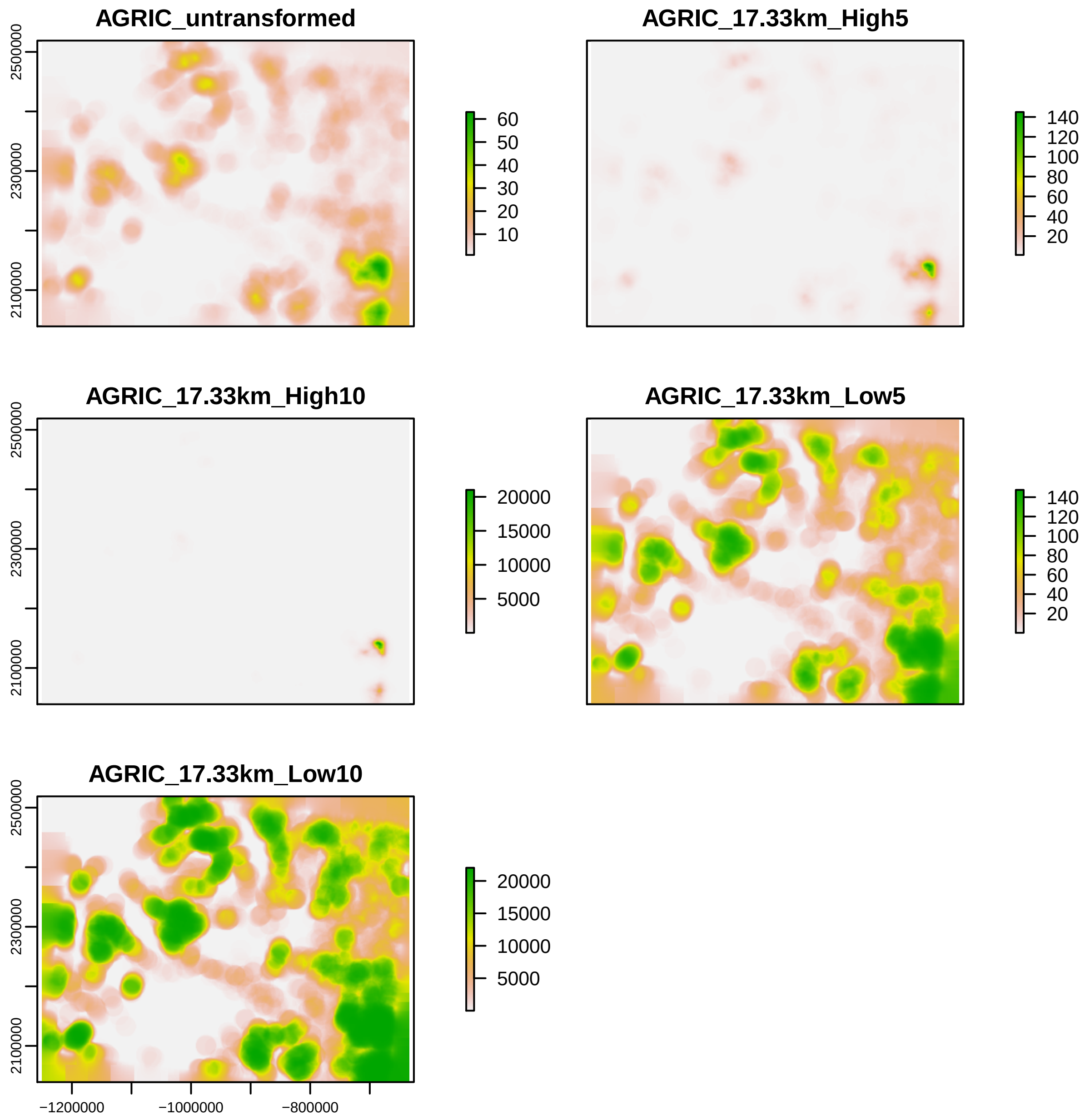
AGRIC_untransformed
AGRIC_17.33km_High5
AGRIC_17.33km_High10
AGRIC_17.33km_Low5
AGRIC_17.33km_Low10
2500000
2300000
2100000
−1200000
−1000000
−800000
60
50
40
30
20
10
140
120
100
80
60
40
20
20000
15000
10000
5000

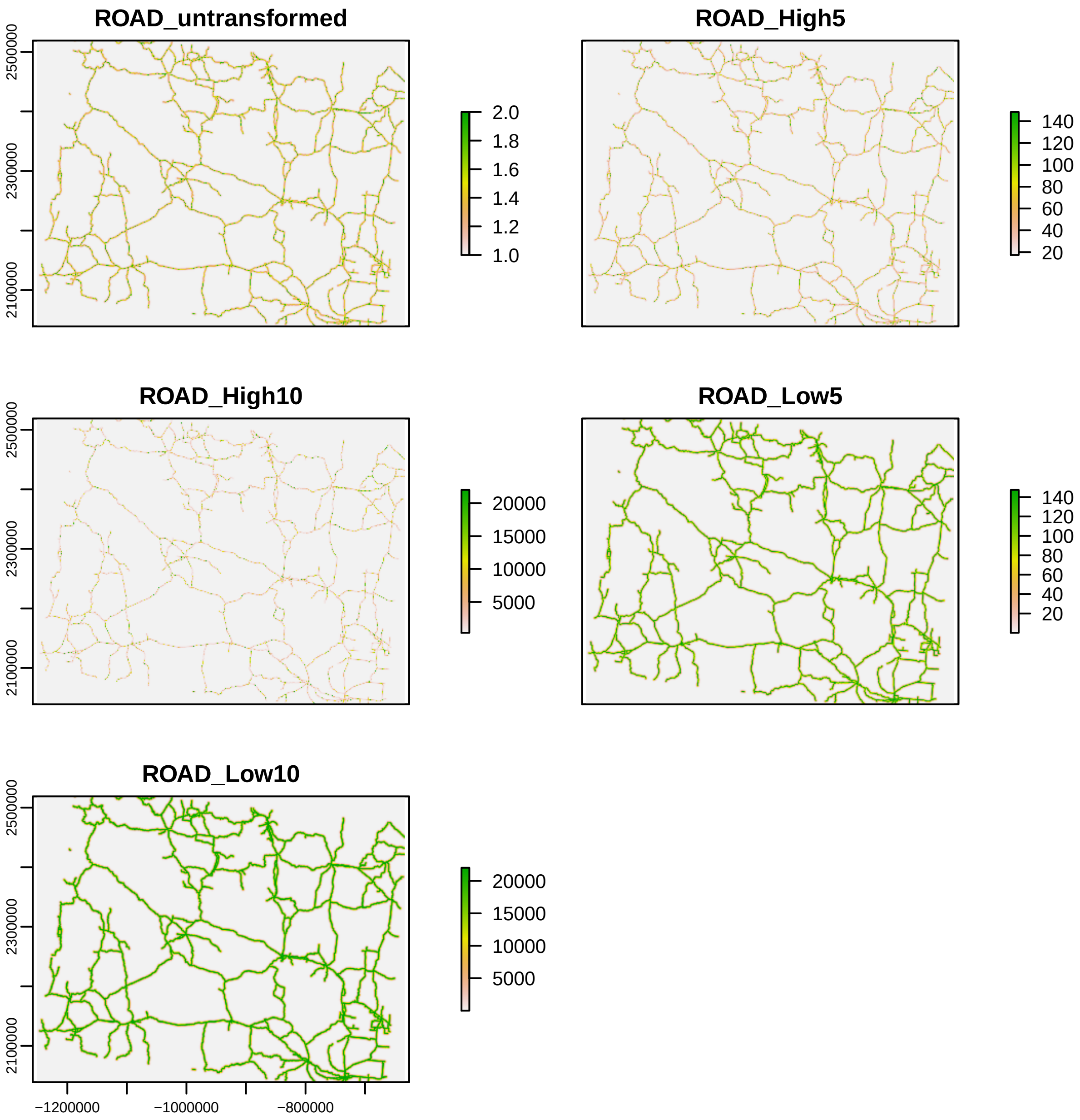
ROAD_untransformed
ROAD_High5
ROAD_High10
ROAD_Low5
ROAD_Low10
2500000
2300000
2100000
−1200000
−1000000
−800000
2.0
1.8
1.6
1.4
1.2
1.0
140
120
100
80
60
40
20
20000
15000
10000
5000
