# Supplementary material for: Landscape characteristics influencing the genetic structure of greater sage-grouse within the stronghold of their range: a holistic modeling approach
Source: Ecol Evol. 2015 May 1;5(10):1955–69. doi: 10.1002/ece3.1479 (PMC4449751; doi:10.1002/ece3.1479)
Supplement: Supplementary file 4 [file ece30005-1955-sd4.docx]

**Figure S1**. Representation of transformations equations used to vary resistance surfaces: a) transformation using equation 1 (high transformation), which places an emphasis on variation in high resistance habitat and homogenizes low resistance habitat, and b) transformation using equation 2 (low transformation) which emphasizes variation in low resistance habitat and homogenizes high resistance habitat. In both cases α was set to 5 for this visualization.

**Figure S2.** Correlation between G_ST_ and Jost D_est_ for 37 lek groupings of sage-grouse across Wyoming.

**Figure S3.** Strength of correlation between pairwise resistances calculated from resistance surfaces transformed using different moving window sizes (scale), and strength transformation (transformation; *high* and *low* transformation using equation 1) or scale and transformation (cross). High values suggest transformations have little effect on the resulting pairwise resistance as they are highly correlated.

**Figure S4.** Correlation between resistance surfaces derived from seasonal habitat suitability modeling.

**Figure S5.** Distribution and correlation between resistance surfaces derived from individual landscape characteristics.

**Figure S6.** Pairwise correlation for model selection criteria describing dispersal hypothesis between lek groupings of sage-grouse across Wyoming.
