# Supplementary material for: Landscape characteristics influencing the genetic structure of greater sage-grouse within the stronghold of their range: a holistic modeling approach
Source: Ecol Evol. 2015 May 1;5(10):1955–69. doi: 10.1002/ece3.1479 (PMC4449751; doi:10.1002/ece3.1479)
Supplement: Supplementary file 5 [file ece30005-1955-sd5.docx]

**Table S1.** Sample size and genetic summary statistics for 37 populations of the Greater Sage-Grouse across Wyoming (Fig 1).

| **Group** | **N** | **AR** | **SD** | **H_obs_** | **SD** | **H_exp_** | **SD** | **F_IS_** | **SD** |
| --- | --- | --- | --- | --- | --- | --- | --- | --- | --- |
| 1 | 10 | 6.36 | 2.08 | 0.78 | 0.19 | 0.76 | 0.09 | -0.03 | 0.21 |
| 2 | 12 | 6.52 | 1.70 | 0.79 | 0.11 | 0.77 | 0.07 | -0.03 | 0.09 |
| 3 | 14 | 6.40 | 1.50 | 0.80 | 0.12 | 0.76 | 0.06 | -0.05 | 0.12 |
| 4 | 18 | 6.51 | 1.68 | 0.75 | 0.13 | 0.79 | 0.09 | 0.05 | 0.14 |
| 5 | 15 | 7.01 | 1.74 | 0.83 | 0.12 | 0.77 | 0.10 | -0.08 | 0.12 |
| 6 | 21 | 7.45 | 1.80 | 0.81 | 0.11 | 0.81 | 0.07 | 0.01 | 0.07 |
| 7 | 17 | 6.74 | 2.07 | 0.76 | 0.13 | 0.78 | 0.09 | 0.03 | 0.10 |
| 8 | 15 | 5.72 | 1.42 | 0.78 | 0.17 | 0.72 | 0.10 | -0.08 | 0.13 |
| 9 | 11 | 6.01 | 1.52 | 0.79 | 0.11 | 0.74 | 0.10 | -0.07 | 0.15 |
| 10 | 56 | 7.09 | 1.81 | 0.81 | 0.09 | 0.82 | 0.07 | 0.02 | 0.07 |
| 11 | 11 | 6.10 | 1.69 | 0.69 | 0.12 | 0.73 | 0.08 | 0.05 | 0.14 |
| 12 | 52 | 6.50 | 1.46 | 0.80 | 0.09 | 0.80 | 0.06 | 0.00 | 0.07 |
| 13 | 12 | 5.91 | 1.87 | 0.75 | 0.16 | 0.74 | 0.11 | -0.02 | 0.19 |
| 14 | 16 | 7.52 | 1.86 | 0.85 | 0.18 | 0.80 | 0.15 | -0.07 | 0.09 |
| 15 | 19 | 6.57 | 1.41 | 0.83 | 0.11 | 0.79 | 0.07 | -0.05 | 0.11 |
| 16 | 10 | 7.04 | 1.91 | 0.86 | 0.13 | 0.78 | 0.07 | -0.11 | 0.17 |
| 17 | 14 | 6.13 | 1.31 | 0.80 | 0.13 | 0.76 | 0.09 | -0.06 | 0.16 |
| 18 | 19 | 6.92 | 1.68 | 0.81 | 0.15 | 0.79 | 0.09 | -0.03 | 0.14 |
| 19 | 28 | 6.53 | 1.72 | 0.80 | 0.16 | 0.78 | 0.13 | -0.01 | 0.11 |
| 20 | 24 | 6.74 | 1.87 | 0.82 | 0.12 | 0.79 | 0.08 | -0.03 | 0.13 |
| 21 | 16 | 6.35 | 1.20 | 0.80 | 0.11 | 0.76 | 0.08 | -0.05 | 0.09 |
| 22 | 10 | 6.90 | 2.15 | 0.84 | 0.12 | 0.78 | 0.07 | -0.08 | 0.10 |
| 23 | 13 | 6.82 | 1.80 | 0.78 | 0.16 | 0.75 | 0.12 | -0.05 | 0.16 |
| 24 | 12 | 5.89 | 1.51 | 0.81 | 0.12 | 0.74 | 0.10 | -0.10 | 0.19 |
| 25 | 22 | 7.15 | 1.59 | 0.80 | 0.09 | 0.81 | 0.07 | 0.01 | 0.08 |
| 26 | 19 | 6.46 | 1.55 | 0.79 | 0.11 | 0.77 | 0.08 | -0.03 | 0.12 |
| 27 | 14 | 5.05 | 1.25 | 0.67 | 0.22 | 0.70 | 0.12 | 0.06 | 0.25 |
| 28 | 14 | 7.04 | 1.37 | 0.80 | 0.16 | 0.80 | 0.06 | 0.02 | 0.15 |
| 29 | 11 | 6.45 | 1.40 | 0.78 | 0.10 | 0.77 | 0.07 | -0.01 | 0.13 |
| 30 | 13 | 6.72 | 1.24 | 0.79 | 0.13 | 0.79 | 0.06 | 0.00 | 0.12 |
| 31 | 20 | 6.26 | 1.47 | 0.80 | 0.11 | 0.78 | 0.09 | -0.04 | 0.12 |
| 32 | 20 | 6.56 | 1.40 | 0.81 | 0.11 | 0.78 | 0.07 | -0.05 | 0.13 |
| 33 | 13 | 6.04 | 1.25 | 0.75 | 0.15 | 0.74 | 0.08 | -0.02 | 0.17 |
| 34 | 11 | 6.44 | 1.64 | 0.78 | 0.12 | 0.76 | 0.09 | -0.03 | 0.17 |
| 35 | 11 | 6.52 | 1.74 | 0.83 | 0.12 | 0.78 | 0.07 | -0.06 | 0.13 |
| 36 | 13 | 7.11 | 1.94 | 0.82 | 0.14 | 0.79 | 0.08 | -0.03 | 0.13 |
| 37 | 29 | 6.78 | 1.51 | 0.79 | 0.09 | 0.80 | 0.06 | 0.01 | 0.12 |

**Table S2.** Table of univariate model selection criteria for habitat suitability models describing functional connectivity for sage-grouse across Wyoming.

| **Var** | **Scale**  **(km)** | **Trans** | **Mantel**  **R** | **R^2^_β_** | **R^2^_GLMM(m)_** | **AIC_c_** | **DIC** | **Mean**  **rank** | **SD**  **rank** |
| --- | --- | --- | --- | --- | --- | --- | --- | --- | --- |
| NEST | 15 | untrans | 0.62 | 0.32 | 0.48 | -4951.56 | -5083.05 | 8.00 | 1.15 |
| NEST | 15 | high5 | 0.64 | 0.34 | 0.53 | -4957.45 | -5087.09 | 5.75 | 1.26 |
| NEST | 15 | high10 | 0.63 | 0.39 | 0.67 | -4959.08 | -5097.04 | 2.50 | 1.00 |
| NEST | 15 | low5 | 0.56 | 0.31 | 0.28 | -4939.63 | -5077.23 | 12.00 | 0.00 |
| NEST | 15 | low10 | 0.53 | 0.31 | 0.23 | -4936.41 | -5076.75 | 13.50 | 1.00 |
| NEST | 644 | untrans | 0.64 | 0.32 | 0.47 | -4952.82 | -5082.01 | 8.25 | 0.50 |
| NEST | 644 | high5 | 0.66 | 0.34 | 0.55 | -4958.80 | -5085.29 | 5.00 | 0.82 |
| NEST | 644 | high10 | 0.67 | 0.41 | 0.69 | -4957.52 | -5091.52 | 3.00 | 2.00 |
| NEST | 644 | low5 | 0.56 | 0.31 | 0.28 | -4939.86 | -5077.49 | 10.75 | 0.50 |
| NEST | 644 | low10 | 0.52 | 0.31 | 0.23 | -4936.00 | -5076.86 | 14.50 | 0.58 |
| NEST | 1733 | untrans | 0.69 | 0.33 | 0.46 | -4957.71 | -5082.24 | 7.25 | 1.71 |
| NEST | 1733 | high5 | 0.73 | 0.38 | 0.60 | -4969.44 | -5086.55 | 3.50 | 1.73 |
| NEST | 1733 | high10 | 0.75 | 0.53 | 0.75 | -4965.35 | -5088.33 | 1.75 | 0.96 |
| NEST | 1733 | low5 | 0.58 | 0.32 | 0.28 | -4941.46 | -5078.08 | 10.25 | 0.50 |
| NEST | 1733 | low10 | 0.52 | 0.31 | 0.23 | -4936.26 | -5077.12 | 14.00 | 0.82 |
| SUMMER | 15 | untrans | 0.48 | 0.30 | 0.40 | -4930.43 | -5071.85 | 11.25 | 2.99 |
| SUMMER | 15 | high5 | 0.49 | 0.30 | 0.44 | -4931.16 | -5072.22 | 9.50 | 3.11 |
| SUMMER | 15 | high10 | 0.43 | 0.30 | 0.56 | -4914.25 | -5067.02 | 11.25 | 5.56 |
| SUMMER | 15 | low5 | 0.51 | 0.31 | 0.24 | -4935.01 | -5075.85 | 8.25 | 2.63 |
| SUMMER | 15 | low10 | 0.51 | 0.31 | 0.22 | -4935.14 | -5076.91 | 7.25 | 5.44 |
| SUMMER | 644 | untrans | 0.52 | 0.30 | 0.41 | -4932.85 | -5071.69 | 9.75 | 2.22 |
| SUMMER | 644 | high5 | 0.52 | 0.30 | 0.44 | -4932.75 | -5071.42 | 9.50 | 3.11 |
| SUMMER | 644 | high10 | 0.47 | 0.30 | 0.57 | -4915.10 | -5064.01 | 10.00 | 5.66 |
| SUMMER | 644 | low5 | 0.54 | 0.31 | 0.26 | -4936.44 | -5075.85 | 6.25 | 2.63 |
| SUMMER | 644 | low10 | 0.52 | 0.31 | 0.22 | -4936.24 | -5077.11 | 5.50 | 5.92 |
| SUMMER | 1733 | untrans | 0.57 | 0.31 | 0.39 | -4937.55 | -5072.77 | 6.75 | 3.30 |
| SUMMER | 1733 | high5 | 0.57 | 0.31 | 0.45 | -4937.68 | -5072.10 | 5.50 | 3.70 |
| SUMMER | 1733 | high10 | 0.53 | 0.32 | 0.59 | -4919.79 | -5062.78 | 7.50 | 7.55 |
| SUMMER | 1733 | low5 | 0.54 | 0.31 | 0.25 | -4936.89 | -5076.15 | 5.75 | 3.59 |
| SUMMER | 1733 | low10 | 0.52 | 0.31 | 0.22 | -4935.99 | -5077.02 | 6.00 | 4.97 |
| WINTER | 15 | untrans | 0.60 | 0.39 | 0.69 | -4969.92 | -5120.76 | 3.25 | 1.50 |
| WINTER | 15 | high5 | 0.62 | 0.35 | 0.57 | -4967.74 | -5101.75 | 8.75 | 1.89 |
| WINTER | 15 | high10 | 0.59 | 0.38 | 0.68 | -4967.65 | -5119.04 | 5.00 | 2.16 |
| WINTER | 15 | low5 | 0.58 | 0.35 | 0.59 | -4964.18 | -5104.93 | 8.75 | 1.26 |
| WINTER | 15 | low10 | 0.56 | 0.33 | 0.52 | -4957.71 | -5096.61 | 12.75 | 0.50 |
| WINTER | 644 | untrans | 0.63 | 0.40 | 0.69 | -4971.93 | -5115.57 | 2.75 | 1.26 |
| WINTER | 644 | high5 | 0.65 | 0.36 | 0.60 | -4970.99 | -5101.93 | 6.25 | 2.22 |
| WINTER | 644 | high10 | 0.63 | 0.41 | 0.71 | -4966.85 | -5117.16 | 3.75 | 2.87 |
| WINTER | 644 | low5 | 0.58 | 0.34 | 0.55 | -4963.07 | -5101.31 | 11.00 | 0.82 |
| WINTER | 644 | low10 | 0.54 | 0.32 | 0.45 | -4954.81 | -5093.45 | 14.25 | 0.50 |
| WINTER | 1733 | untrans | 0.62 | 0.38 | 0.67 | -4971.63 | -5113.18 | 4.75 | 1.89 |
| WINTER | 1733 | high5 | 0.64 | 0.36 | 0.60 | -4969.68 | -5101.04 | 8.00 | 2.45 |
| WINTER | 1733 | high10 | 0.64 | 0.41 | 0.72 | -4965.04 | -5115.45 | 4.00 | 3.83 |
| WINTER | 1733 | low5 | 0.58 | 0.33 | 0.49 | -4963.45 | -5099.95 | 12.00 | 0.82 |
| WINTER | 1733 | low10 | 0.55 | 0.33 | 0.36 | -4954.06 | -5092.36 | 14.75 | 0.50 |

**Table S3.** Table of univariate model selection criteria for individual landscape variables describing functional connectivity for sage-grouse across Wyoming.

| **Var** | **Scale**  **(km)** | **Trans** | **Mantel**  **R** | **R^2^_β_** | **R^2^_GLMM(m)_** | **AIC_c_** | **DIC** | **Mean**  **rank** | **SD**  **rank** |
| --- | --- | --- | --- | --- | --- | --- | --- | --- | --- |
| FOR | 15 | untrans | 0.35 | 0.26 | 0.51 | -4882.58 | -5037.24 | 5.50 | 3.00 |
| FOR | 15 | high5 | 0.54 | 0.32 | 0.27 | -4946.55 | -5086.10 | 4.00 | 4.08 |
| FOR | 15 | high10 | 0.53 | 0.32 | 0.33 | -4948.30 | -5087.47 | 3.25 | 3.86 |
| FOR | 15 | low5 | 0.33 | 0.15 | 0.35 | -4792.63 | -4932.63 | 9.50 | 1.00 |
| FOR | 15 | low10 | 0.37 | 0.09 | 0.13 | -4757.62 | -4891.10 | 12.00 | 1.41 |
| FOR | 644 | untrans | 0.45 | 0.25 | 0.49 | -4873.30 | -5015.61 | 6.50 | 3.00 |
| FOR | 644 | high5 | 0.58 | 0.32 | 0.39 | -4946.66 | -5081.78 | 3.50 | 1.73 |
| FOR | 644 | high10 | 0.52 | 0.30 | 0.45 | -4930.34 | -5069.01 | 4.75 | 0.50 |
| FOR | 644 | low5 | 0.42 | 0.09 | 0.20 | -4755.51 | -4885.54 | 11.50 | 0.58 |
| FOR | 644 | low10 | 0.42 | 0.06 | 0.11 | -4735.63 | -4865.93 | 15.00 | 0.00 |
| FOR | 1733 | untrans | 0.37 | 0.18 | 0.39 | -4815.48 | -4955.64 | 8.50 | 1.00 |
| FOR | 1733 | high5 | 0.53 | 0.31 | 0.42 | -4939.71 | -5078.38 | 4.25 | 0.50 |
| FOR | 1733 | high10 | 0.44 | 0.27 | 0.46 | -4894.19 | -5038.02 | 5.25 | 1.50 |
| FOR | 1733 | low5 | 0.35 | 0.08 | 0.17 | -4747.96 | -4880.51 | 12.75 | 0.50 |
| FOR | 1733 | low10 | 0.36 | 0.08 | 0.16 | -4745.98 | -4878.20 | 13.75 | 0.50 |
| SAGE | 15 | untrans | 0.57 | 0.32 | 0.26 | -4939.05 | -5076.64 | 8.50 | 0.58 |
| SAGE | 15 | high5 | 0.61 | 0.31 | 0.43 | -4943.83 | -5075.99 | 9.00 | 4.97 |
| SAGE | 15 | high10 | 0.58 | 0.33 | 0.58 | -4938.81 | -5075.75 | 7.00 | 4.24 |
| SAGE | 15 | low5 | 0.53 | 0.31 | 0.23 | -4936.19 | -5076.82 | 10.50 | 2.38 |
| SAGE | 15 | low10 | 0.50 | 0.31 | 0.22 | -4935.28 | -5076.94 | 11.75 | 5.85 |
| SAGE | 644 | untrans | 0.58 | 0.32 | 0.27 | -4939.53 | -5076.71 | 7.50 | 0.58 |
| SAGE | 644 | high5 | 0.63 | 0.32 | 0.46 | -4945.93 | -5075.75 | 6.50 | 4.43 |
| SAGE | 644 | high10 | 0.61 | 0.34 | 0.60 | -4939.59 | -5073.77 | 6.00 | 5.66 |
| SAGE | 644 | low5 | 0.53 | 0.31 | 0.23 | -4936.36 | -5076.89 | 9.00 | 3.37 |
| SAGE | 644 | low10 | 0.50 | 0.31 | 0.22 | -4935.30 | -5076.96 | 10.75 | 5.85 |
| SAGE | 1733 | untrans | 0.59 | 0.32 | 0.27 | -4940.55 | -5076.88 | 5.75 | 0.96 |
| SAGE | 1733 | high5 | 0.70 | 0.33 | 0.49 | -4954.47 | -5076.30 | 4.50 | 3.87 |
| SAGE | 1733 | high10 | 0.71 | 0.42 | 0.67 | -4952.70 | -5073.35 | 4.75 | 6.85 |
| SAGE | 1733 | low5 | 0.54 | 0.31 | 0.24 | -4937.36 | -5076.87 | 8.75 | 1.89 |
| SAGE | 1733 | low10 | 0.51 | 0.31 | 0.22 | -4935.41 | -5076.96 | 9.75 | 5.85 |
| RUGG | 0 | untrans | 0.57 | 0.32 | 0.28 | -4944.64 | -5082.36 | 13.25 | 1.71 |
| RUGG | 0 | high5 | 0.58 | 0.32 | 0.29 | -4947.02 | -5084.02 | 10.75 | 1.89 |
| RUGG | 0 | high10 | 0.61 | 0.33 | 0.37 | -4954.94 | -5088.67 | 8.00 | 2.94 |
| RUGG | 0 | low5 | 0.47 | 0.35 | 0.68 | -4923.12 | -5103.32 | 5.75 | 6.24 |
| RUGG | 0 | low10 | 0.47 | 0.35 | 0.68 | -4926.05 | -5105.46 | 5.25 | 5.91 |
| RUGG | 644 | untrans | 0.58 | 0.32 | 0.29 | -4944.92 | -5081.77 | 13.25 | 2.36 |
| RUGG | 644 | high5 | 0.62 | 0.33 | 0.39 | -4954.66 | -5087.19 | 8.75 | 2.50 |
| RUGG | 644 | high10 | 0.63 | 0.34 | 0.54 | -4960.41 | -5091.50 | 5.00 | 2.71 |
| RUGG | 644 | low5 | 0.60 | 0.33 | 0.54 | -4954.60 | -5088.42 | 7.00 | 1.41 |
| RUGG | 644 | low10 | 0.60 | 0.32 | 0.46 | -4951.43 | -5084.33 | 9.50 | 2.38 |
| RUGG | 1733 | untrans | 0.58 | 0.32 | 0.29 | -4945.08 | -5082.01 | 12.50 | 2.38 |
| RUGG | 1733 | high5 | 0.62 | 0.33 | 0.42 | -4957.00 | -5088.85 | 6.75 | 2.63 |
| RUGG | 1733 | high10 | 0.61 | 0.35 | 0.58 | -4958.54 | -5092.78 | 4.25 | 1.50 |
| RUGG | 1733 | low5 | 0.54 | 0.36 | 0.69 | -4931.72 | -5097.95 | 4.50 | 5.74 |
| RUGG | 1733 | low10 | 0.54 | 0.36 | 0.67 | -4939.49 | -5096.11 | 5.50 | 4.43 |
| AGRIC | 15 | untrans | 0.51 | 0.32 | 0.55 | -4935.29 | -5079.17 | 3.25 | 2.06 |
| AGRIC | 15 | high5 | 0.61 | 0.32 | 0.32 | -4944.40 | -5078.75 | 3.75 | 4.86 |
| AGRIC | 15 | high10 | 0.61 | 0.31 | 0.43 | -4941.07 | -5072.95 | 5.00 | 2.16 |
| AGRIC | 15 | low5 | 0.36 | 0.25 | 0.54 | -4794.49 | -4945.95 | 9.00 | 2.00 |
| AGRIC | 15 | low10 | 0.31 | 0.16 | 0.33 | -4730.97 | -4866.38 | 11.75 | 1.50 |
| AGRIC | 644 | untrans | 0.49 | 0.29 | 0.59 | -4884.15 | -5030.87 | 6.00 | 2.71 |
| AGRIC | 644 | high5 | 0.60 | 0.31 | 0.39 | -4944.13 | -5077.53 | 4.25 | 3.20 |
| AGRIC | 644 | high10 | 0.57 | 0.31 | 0.52 | -4935.83 | -5071.85 | 5.00 | 1.41 |
| AGRIC | 644 | low5 | 0.37 | 0.08 | 0.20 | -4742.70 | -4873.95 | 11.75 | 0.50 |
| AGRIC | 644 | low10 | 0.28 | 0.02 | 0.04 | -4708.94 | -4841.74 | 15.00 | 0.00 |
| AGRIC | 1733 | untrans | 0.55 | 0.29 | 0.55 | -4836.81 | -4967.58 | 7.75 | 2.50 |
| AGRIC | 1733 | high5 | 0.57 | 0.31 | 0.56 | -4921.68 | -5058.02 | 5.25 | 1.50 |
| AGRIC | 1733 | high10 | 0.50 | 0.30 | 0.60 | -4847.53 | -4992.70 | 6.00 | 3.37 |
| AGRIC | 1733 | low5 | 0.49 | 0.07 | 0.13 | -4743.01 | -4870.38 | 12.25 | 0.96 |
| AGRIC | 1733 | low10 | 0.41 | 0.05 | 0.06 | -4728.56 | -4860.49 | 14.00 | 0.00 |
| ROAD | 0 | untrans | 0.51 | 0.31 | 0.23 | -4936.13 | -5077.31 | 3.50 | 1.00 |
| ROAD | 0 | high5 | 0.53 | 0.32 | 0.25 | -4938.49 | -5078.55 | 2.50 | 1.00 |
| ROAD | 0 | high10 | 0.56 | 0.32 | 0.31 | -4945.42 | -5082.90 | 1.50 | 1.00 |
| ROAD | 0 | low5 | 0.27 | 0.28 | 0.54 | -4898.97 | -5065.82 | 3.50 | 1.00 |
| ROAD | 0 | low10 | 0.19 | 0.28 | 0.57 | -4876.29 | -5057.30 | 4.00 | 2.00 |
